# Ultrathin Phosphate‐Modulated Co Phthalocyanine/g‐C_3_N_4_ Heterojunction Photocatalysts with Single Co–N_4_ (II) Sites for Efficient O_2_ Activation

**DOI:** 10.1002/advs.202001543

**Published:** 2020-06-25

**Authors:** Xiaoyu Chu, Yang Qu, Amir Zada, Linlu Bai, Zhijun Li, Fan Yang, Lina Zhao, Guiling Zhang, Xiaojun Sun, Zhao‐Di Yang, Liqiang Jing

**Affiliations:** ^1^ Key Laboratory of Functional Inorganic Materials Chemistry School of Chemistry and Material Sciences Heilongjiang University Harbin Heilongjiang 150080 China; ^2^ School of Chemical and Environmental Engineering Harbin University of Science and Technology Harbin Heilongjiang 150080 China; ^3^ Department of Food and Environmental Engineering East University of Heilongjiang Harbin Heilongjiang 150080 China; ^4^ Department of Chemistry Abdul Wali Khan University Mardan 23200 Pakistan

**Keywords:** charge transfer and separation, Co phthalocyanine/g‐C_3_N_4_ heterojunctions, phosphate‐modulated H‐bonding interfaces, photocatalytic O_2_ activation, single Co–N_4_ sites

## Abstract

Realization of solar‐driven aerobic organic transformation under atmospheric pressure raises the great challenge for efficiently activating O_2_ by tailored photocatalysts. Guided by theoretical calculation, phosphate groups are used to induce the construction of ultrathin Co phthalocyanine/g‐C_3_N_4_ heterojunctions (CoPc/P‐CN, ≈4 nm) via strengthened H‐bonding interfacial connection, achieving an unprecedented 14‐time photoactivity improvement for UV–vis aerobic 2,4‐dichlorophenol degradation compared to bulk CN by promoted activation of O_2_. It is validated that more ^•^O_2_^−^ radicals are produced through the improved photoreduction of O_2_ by accelerated photoelectron transfer from CN to the ligand of CoPc and then to the abundant single Co–N_4_ (II) catalytic sites, as endowed by the matched dimension, intimate interface even at the molecular level, and high CoPc dispersion of resulted heterojunctions. Interestingly, CoPc/P‐CN also exhibits outstanding photoactivities in the aerobic oxidation of aromatic alcohols. This work showcases a feasible route to realize efficient photocatalytic O_2_ activation by exploiting the potential of ultrathin metal phthalocyanine (MPc) assemblies with abundant single‐atom sites. More importantly, a universal facile strategy of H‐bonding‐dominating construction of MPc‐involved heterojunctions is successfully established.

## Graphical abstract

Ultrathin phosphate‐modulated Co phthalocyanine/g‐C_3_N_4_ heterojunctions are successfully fabricated for effective photocatalytic O_2_ activation via phosphate‐induced strengthened H‐bonding interfacial connection, as driven by density functional theory calculation. The accelerated charge kinetics from g‐C_3_N_4_ to the ligand of CoPc and then to the abundant single Co–N_4_ (II) catalytic sites for O_2_ is evidenced to contribute to the outstanding photocatalytic performances.

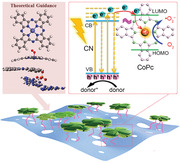

## 1 Introduction

Solar‐driven organic synthesis with oxidative transformation as representative to high‐value chemicals has exhibited strong confidence in science and industry to be competitive with traditional catalytic processes.^[^ ^1^, ^2^, ^3^ ^]^ Especially, to use O_2_ as the oxidant at atmospheric pressure is a much greener choice. While considering O_2_ reduction is an electron spin‐forbidden process and kinetically unflavored,^[^ ^4^, ^5^ ^]^ the critical challenge of obtaining high photocatalytic performances lies on how to tailor the photocatalyst to efficiently activate O_2_ under light irradiation. Among all semiconductors, graphic carbon nitride (CN) has a high energy level of conduction band (CB) naturally favoring the photoreduction of O_2_, which makes CN an ideal candidate along with the metal‐free, nontoxic, visible‐light responsive, robust and low‐cost features.^[^ ^6^, ^7^, ^8^ ^]^ However, bulk CN suffers from its intrinsic poor charge separation, small specific surface area, and insufficient visible‐light absorption, significantly retarding the photocatalytic efficiency. The 2D morphology control to fabricate ultrathin CN nanosheets could shorten the charge diffusion length from the bulk to surface then to inhibit the charge recombination, meanwhile enlarge the specific surface area. To further improve the charge separation, one of the most common strategy is to couple CN nanosheets with another semiconductor to construct heterojunctions.^[^ ^9^, ^10^ ^]^ Whereas, widely employed spherical or rod‐like semiconductors as the coupler always results in poor dimension‐matching, raising the necessity of 2D coupler, to the best, with visible‐light extension.^[^ ^11^ ^]^ Nevertheless, favorable charge separation by constructing 2D/2D CN‐based heterojunctions is only the foundation. The inevitable key to markedly activate O_2_ is to introduce indispensable active catalytic sites for O_2_.

Based on all above consideration, the coupler choice turns to the metal phthalocyanine (MPc) as photosensitizer by mimicking the natural photosynthesis, whose study is undergoing rapid development.^[^ ^12^, ^13^, ^14^, ^15^ ^]^ MPc is a macrocyclic molecule with a planar conjugated array of 18‐p electrons, and easily aggregate to a 2D assembly due to strong *π*–*π* stacking interaction.^[^ ^12^, ^13^ ^]^ Generally, they are mainly utilized to extend the visible‐light absorption in photocatalytic applications.^[^ ^13^, ^14^ ^]^ What's always neglected is that MPcs themselves are organic semiconductors with an intrinsic lowest unoccupied molecular orbital (LUMO) and highest unoccupied molecular orbital (HOMO).^[^ ^16^ ^]^ Especially, the energy level of LUMO of MPc is close or slightly higher to the CB bottom of CN, hence it is highly possible to realize the photogenerated electron transfer by constructing dimension‐matched MPc/CN heterojunctions for significantly accelerated charge transfer kinetics meanwhile extended visible‐light absorption. Noteworthily, the MPc molecule possesses well‐defined single‐atom metal site with the coordination of four N atoms, as noted M–N_4_. Interestingly, it resembles one of the mostly investigated heterogeneous single‐atom M–N–C catalytic sites, as obtained by elaborately anchoring single metal atoms on N‐doped carbon materials or carbon nitride.^[^ ^17^, ^18^, ^19^ ^]^ It is well known that single‐atom catalysis is one of the hottest research fronts, and typical M–N–C catalysts have been reported to exhibit dramatic catalytic performances by activating the reactant molecules like CO_2_ and H_2_O, in the electrochemical/chemical conversions.^[^ ^17^, ^18^, ^19^ ^]^ Analogously, M–N_4_ sites of MPc with homogeneous configuration than M–N–C sites, as exposed on the external surfaces of MPc assemblies supported on CN, might function as uniform, effective, and stable single‐atom catalytic sites for photocatalytic O_2_, unfortunately which is seldom emphasized in photocatalysis so far and lacks rational structure–function correlation. Therefore, 2D/2D MPc/CN heterojunctions are first proposed to activate O_2_ using single M–N_4_ sites for efficient photocatalytic oxidative transformation.

Attention is then directed to how to obtain efficient MPc/CN heterojunctions by controllably assembling MPc onto CN with high dispersion and proper thickness to guarantee as much as exposed single M–N_4_ sites, greatly reduced the charge transfer resistance and favorable stability. Accordingly, a refined synthetic strategy to precisely modulate the MPc/CN interfaces at the molecular level is urged to be developed. Or rather, the interactions between MPc assemblies and CN nanosheets should be substantially strengthened to rival the *π*–*π* interaction between MPc molecules themselves, principally depending on the interfacial interaction mode. For the heterogeneous MPc‐involved heterojunctions, versatile covalent and noncovalent interaction modes have already been proposed.^[^ ^20^, ^21^, ^22^ ^]^ By contrast, the noncovalent one is preferable mainly owing to the facile synthesis and ease to scale up. Enlightened by the previous study, the N atoms in MPc could interact with hydroxyl groups in the solvent molecules like water to form H‐bonding.^[^ ^23^ ^]^ Thus, it is reasonable to infer MPc might interact with the surface hydroxyl groups of solid like CN through analogical H‐bonding effect as an optional interaction mode, which had already been verified in our work on ZnPc/BiVO_4_ nanocomposites.^[^ ^16^ ^]^ Therefore, it is hypothesized to increase surface hydroxyl amount of CN by premodified inorganic acids like phosphate to strengthen the interfacial H‐bonding connection might induce highly dispersed and thickness‐controlled MPc assemblies on CN nanosheets.

In order to implement the synthetic strategy more efficiently by avoiding the traditional “try and see” on account of above analysis, the theoretical calculation‐guided rational design in advance of practical experiments comes into our sight. The optimum MPc candidate has first been screened by calculating and comparing the adsorption energies of O_2_ on the widely investigated MPcs. Subsequently the typical phosphate modulation for CoPc/CN heterojunctions (CoPc/P‐CN) through forming H‐bonding connection could be modeled and then to be evaluated on the feasibility. As this protocol, here, density functional theory (DFT) method was applied for all calculations. For reducing the degrees of freedom and lowering the required computing time, periodical g‐C_3_N_4_ was simplified as a model structure with three melem units and one hydroxyl group on C atom.

**Figure 1:**
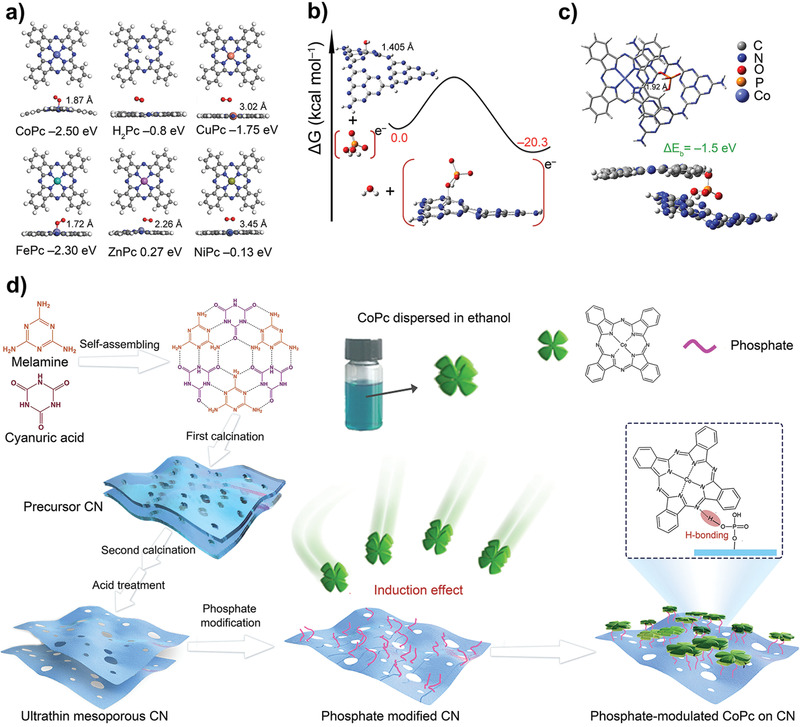
a) Comparison of O_2_ adsorption energies on different phthalocyanines. b) The free energy of C–O–P formation in the condensation reaction between H_2_PO_4_^−^ and CN. c) The optimized geometrical structure for CoPc/P‐CN and relevant binding energy. d) Synthetic illustration of the ultrathin CoPc/P‐CN nanocomposites.

For the hydroxyl‐CN, the adsorption energy of O_2_ was calculated to be −1.76 eV (Figure S1, Supporting Information). Subsequently, the adsorption energies of O_2_ on series of common phthalocyanines CoPc (II), FePc (II), CuPc (II), ZnPc (II), NiPc (II), and H_2_Pc were calculated (**Figure** **1**a), to be −2.50, −2.30, −1.75, 0.27, −0.13, and −0.80 eV, respectively. Obviously, CoPc exhibits the best affinity with O_2_ over other phthalocyanines and hydroxyl‐CN, hence is selected to be the MPc candidate. Next, to inspect the assumed induction effect of phosphate groups, the theoretical model of phosphate modified CN (P‐CN) was built as Figure 1b. By losing one molecule water, an O—P bond forms between the hydroxyl‐CN and NaH_2_PO_4_ as the phosphate source. The free energy difference (Δ*G*) for this process is −20.3 kcal mol^−1^, indicating it is an exothermic process and the formation of P‐CN is thermodynamically permitted. The final stable interaction between one CoPc molecule and P‐CN is confirmed to be through the H‐bonding (N atom of CoPc with the H atom of as‐modified phosphate hydroxyl) effect. As shown in Figure 1c, the calculated binding energy (Δ*E*_b_) for the formed H‐bonding is −1.5 eV, larger than −0.91 eV between two CoPc molecules (Figure S2a, Supporting Information). Comparably, the Δ*E*_b_ between CoPc and hydroxyl‐CN through similar H‐bonding is −1.7 eV (Figure S2b, Supporting Information) while that for CoPc and CN as *π*–*π* interaction mode is −1.5 eV (Figure S2c, Supporting Information). Therefore, by comparing three similar Δ*E*_b_ it is proved H‐bonding could compete with *π*–*π* interaction as possible interaction mode and the modulation effect of phosphates has theoretical feasibility. More abundant hydroxyl groups introduced by phosphates lead to stronger interfacial interaction resulting in higher dispersion of CoPc assemblies. Accordingly, the synthetic paradigm of phosphate modulated CoPc on CN was performed in Figure 1d. First, CN nanosheets were synthesized with melamine and cyanuric acid as the raw material trough H‐bonding self‐assembling process with subsequent two‐step calcination at desired temperature/period, followed by an acid treatment. As‐fabricated CN nanosheets were then dipped in the aqueous solution of NaH_2_PO_4_ to fully get modified with phosphates through a wet chemical process. Finally, the phosphate modified CN was introduced in the ethanol‐dispersed solution of CoPc to finally obtain the CoPc/P‐CN heterojunctions. The thermogravimetric (TG) analysis indicates under the synthetic conditions, the chemical structure of CoPc would be well preserved (Figure S3, Supporting Information).

## 2 Structural Characterization and Photocatalytic Performances

**Figure 2:**
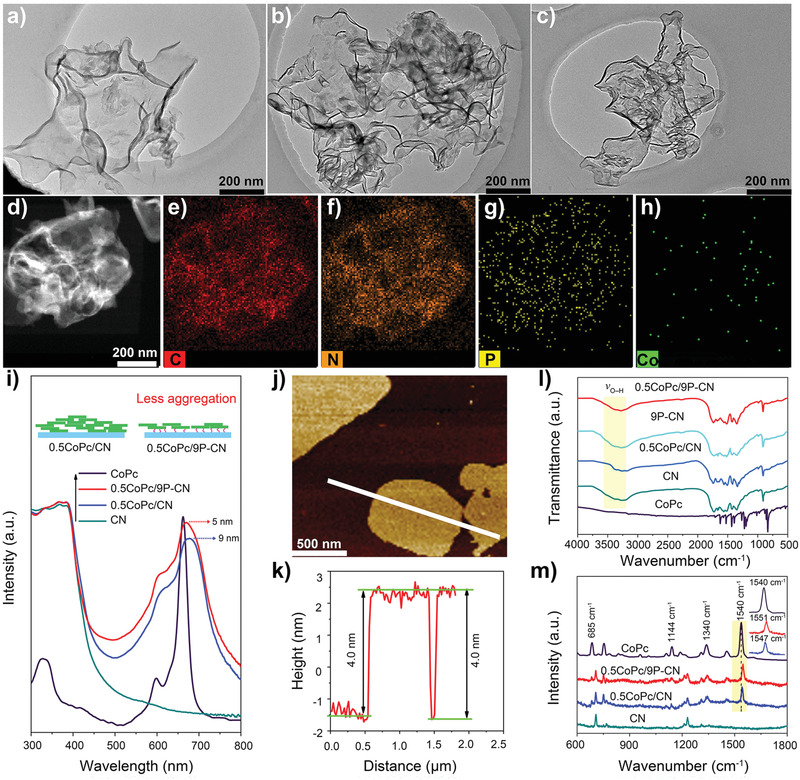
TEM images of a) CN, b) 0.5CoPc/CN, and c) 0.5CoPc/9P‐CN, respectively. d) HAADF‐STEM image of 0.5CoPc/9P‐CN and e–h) the corresponding EDX mapping images of elemental C, N, P, and Co. i) UV–vis absorption spectra of CoPc, CN, 0.5CoPc/CN, and 0.5CoPc/9P‐CN, respectively. j) AFM image and k) the corresponding height profiles of 0.5CoPc/9P‐CN. l) Normalized FT‐IR and m) Raman spectra of CoPc, CN, 0.5CoPc/CN, and 0.5CoPc/9P‐CN, respectively.

The characteristic diffraction peaks at 27.3° and 13.1° of CN nanosheets are ascribed to the facet (002) and (100), respectively (Figure S4a, Supporting Information),^[^ ^7^, ^8^ ^]^ and no typical ones from CoPc appear in the patterns of XCoPc/CN and 0.5CoPc/YP‐CN by either increasing the loading amounts of CoPc or phosphate (X = 0.05–1% and Y = 3–12% by weight), due to the tiny amount and/or high dispersion of CoPc for all samples (Figure S4b,c, Supporting Information). The introduction of CoPc slightly decreases the specific surface area of CN and 9P‐CN (Figure S5, Supporting Information). The high‐resolution transmission electron microscopy (TEM) image of CN (**Figure** **2**a) illustrates silky semitransparent 2D morphology, indicating the successful fabrication of ultrathin CN nanosheets. For CoPc‐introduced ones with 0.5CoPc/CN as a representative (Figure 2b), CN is observed to be covered by fuzzy and rough dark shadows instead of obvious aggregates, which are assigned to the loaded CoPc. In comparison, less and lightened shadows appear on 0.5CoPc/9P‐CN (Figure 2c), implying a high dispersion of uniform CoPc assemblies when phosphates exist. The energy‐dispersive X‐ray (EDX) elemental mapping images (Figure 2d–h) confirm a homogeneous distribution of Co and P elements. As for the X‐ray photoelectron spectroscopy (XPS) spectra (Figure S6, Supporting Information), 0.5CoPc/9P‐CN shows two Co2p peaks at 795.6 and 780.6 eV associated with Co 2p_1/2_ and Co 2p_3/2_ transitions, respectively.^[^ ^24^ ^]^ Combining with the P 2p peak at 133.8 eV, it indicates the successful introduction of CoPc and phosphates, consistent with the EDX results.^[^ ^25^ ^]^ The UV–vis absorption spectra were collected to reveal the existential state of as‐modified CoPc and the light absorption properties (Figure 2i; Figure S7, Supporting Information). The typical spectroscopy of CoPc (dissolved in DMF as monomer) displays two absorbance bands, the sharp Q bands (550–750 nm) attributed to the a_1u_ (*π*)–e_g_ (π*) transition from HOMO to LUMO and the B band (250–350 nm) as a result of the deeper a_2u_ (*π*) to LUMO transition.^[^ ^15^, ^21^ ^]^ For XCoPc/CN, the visible‐light absorption of CN is gradually extended by increasing the CoPc amount (Figure S7a, Supporting Information), simultaneously showing broadened and red‐shift Q bands. In detail, it is demonstrated the red‐shifts of 0.5CoPc/CN are 9 nm compared to those for CoPc monomer. By contrast, for 0.5CoPc/9P‐CN, broader Q bands with smaller red‐shifts of 5 nm are observed with further extended visible‐light absorption. As examined the phosphate modification could not contribute to the absorption of extra visible light (Figure S7b, Supporting Information). Therefore, it is suggested the modified phosphates do induce higher dispersion of CoPc then to obtain the extended visible‐light absorption, indicative of the stronger interfacial H‐bonding interaction as predicted by the theoretical calculation. More importantly, the absorption band features of phthalocyanines could provide additional profound information on the status of supported CoPc. The spectra change like broadened and red‐shifted Q bands of CoPc in the nanocomposites is mainly attributed to J‐aggregates like the brickwork arrangement.^[^ ^26^, ^27^ ^]^ Smaller red‐shifts of Q bands indicate the nature of highly dispersed CoPc induced by the phosphates, or rather looser and thinner J‐aggregates, which is illustrated as the inset scheme in Figure 2i. Additionally, the effect of phosphates is further verified by the absorption of 0.5CoPc/YP‐CN samples (Figure S7c, Supporting Information). With fixed CoPc amount, increasing phosphates bring about broader and smaller red‐shifts of Q bands accompanying gradual extended visible‐light absorption. In an effort to discriminate the thickness of CoPc assemblies in the form of J‐aggregate, the samples CN, 0.5CoPc/CN, and 0.5CoPc/9P‐CN were taken out of ultra high vacuum (UHV) to the ambient environment where atomic force microscope (AFM) imaging was performed at multiple locations across the samples, respectively. The average thickness of CN nanosheets is determined to be 3.4 nm (Figure S8a,b, Supporting Information). In comparison, the average thicknesses of 0.5CoPc/CN and 0.5CoPc/9P‐CN are 4.4 nm (Figure S8c,d, Supporting Information) and 4.0 nm (Figure 2j,k), with 1.0 and 0.6 nm increments, respectively, which firmly evidences the phosphates have induced thinner CoPc assemblies in good agreement with the UV–vis absorption results in Figure 2i. Furthermore, Fourier transform infrared (FT‐IR) and Raman spectra were conducted to clearly validate the interfacial interaction of proposed H‐bonding according to the DFT calculation. In Figure 2l, CN demonstrates the characteristic peaks. Noteworthily, the broad peak located at 3000–3500 cm^−1^ mainly derives from the surface hydroxyl groups.^[^ ^8^ ^]^ For 0.5CoPc/CN and 0.5CoPc/9P‐CN, no characteristic peaks belonging to CoPc are observed due to the tiny CoPc amounts while the peaks of CN are well preserved, suggesting the loaded CoPc interact with CN and P‐CN via the noncovalent bonding. It is noted that the broad peak (3000–3500 cm^−1^) area of 0.5CoPc/CN is smaller than that of pristine CN. For 9P‐CN the introduction of phosphates further increases the broad peak area of CN, whereas the subsequent loading CoPc obviously decreases the area. On the basis of above, it is inferred that the surface hydroxyl groups are significantly involved in the interfacial interaction, in accordance with the predicted H‐bonding mode.

Notably, the characteristic Raman bands and corresponding assignments of pure CoPc consistent with the reference (Figure 2m). For CN, there are two typical bands at 705 and 1231 cm^−1^, both due to the ring breathing modes of s‐triazine.^[^ ^28^ ^]^ For 0.5CoPc/CN and 0.5CoPc/9P‐CN, the bands of CN are maintained, however several bands assigned to CoPc exhibit obvious changes. In particular, the bands at 685 and 1144 cm^−1^ belong to the macrocycle and pyrrole breathing vibrations of CoPc, respectively, as recognized to be sensitive to the intermolecular interactions.^[^ ^29^ ^]^ Interestingly, these bands nearly disappear when CoPc is loaded on CN or P‐CN, strongly implying the weakened intermolecular interaction of CoPc molecules. More noticeably, the bands assigned to C–N–C in CoPc molecule for 0.5CoPc/CN and 0.5CoPc/9P‐CN shift to ≈1547 and 1551 cm^−1^, respectively, compared with 1540 cm^−1^ for pure CoPc, indicating the interfacial interaction substantially might lead to electron density change of C–N to different extents. ^[^ ^29^, ^30^ ^]^ Therefore, both FT‐IR and Raman results coincide with the predicted H‐bonding between the N atom of CoPc ligand and H atom of phosphate hydroxyl. Here, it is worth noting that for 0.5CoPc/CN, H‐bonding interaction is proved to also exist although which is usually reported to be *π*–*π* stacking. This finding agrees well with the as‐calculated similar Δ*E*_b_ of both interaction modes. Moreover, a bit larger red‐shift of 0.5CoPc/9P‐CN than 0.5CoPc/CN is possibly because more abundant hydroxyl groups from the phosphates lead to the stronger H‐bonding interaction. In consequence, combining all above characterization results, it is clearly validated that the strategy of using phosphates to induce highly dispersed ultrathin CoPc assemblies on CN nanosheets via the strengthened H‐bonding connection has been successfully developed.

**Figure 3:**
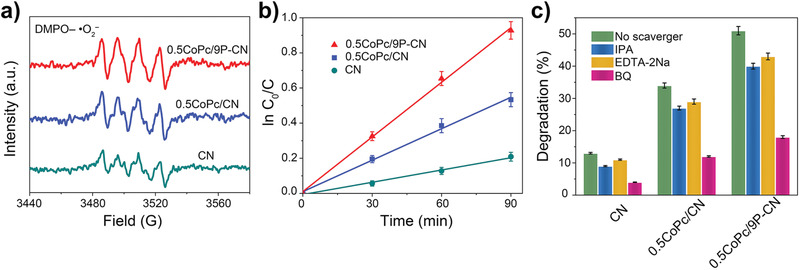
a) DMPO spin‐trapping EPR spectra recorded for ^•^O_2_^−^ radicals. b) Photocatalytic activities for 2,4‐DCP degradation of CN, 0.5CoPc/CN, and 0.5CoPc/9P‐CN under visible‐light irradiation. Data are presented as the mean ± standard deviation (SD) from three independent experiments. c) Photocatalytic activities for 2,4‐DCP degradation of CN, 0.5CoPc/CN, and 0.5CoPc/9P‐CN, respectively, under visible‐light irradiation in the presence of different scavengers. Data are presented as the mean ± SD from three independent experiments.

As the original design, CoPc/P‐CN heterojunctions are capable of activating O_2_ to produce active species more effectively under light irradiation. Here, electron paramagnetic resonance (EPR) technique is applied to discriminate and quasi‐quantify the short‐lived oxidative reactive species, where 5,5‐dimethyl‐1‐pyrroline *N*‐oxide (DMPO) was used to trap the ^•^O_2_^−^ radicals. The EPR spectra were collected for CN, 0.5CoPc/CN, and 0.5CoPc/9P‐CN in the typical reaction solutions under the visible‐light irradiation. **Figure** **3**a shows that during the same period as‐produced amount of ^•^O_2_^−^ increases as the order CN < 0.5CoPc/CN < 0.5CoPc/9P‐CN, indicating 0.5CoPc/9P‐CN possesses the strongest ability to activate O_2_ by photoreduction. Rationally, the as‐produced amount of ^•^O_2_^−^ radicals could directly determine the photoactivity. Moreover, the photocatalytic performances were studied with the common aerobic 2,4‐dichlorophenol (DCP) degradation as a typical reaction. As expected, the photocatalytic degradation rates of 2,4‐DCP are proportional to the amount of ^•^O_2_^−^ radicals (Figure 3b). The scavenger experiments were then performed under the identical conditions by adding 1,4‐benzoquinone (BQ), isopropyl alcohol (IPA), and disodium ethylenediaminetetraacetate (EDTA), corresponding to reactive species ^•^O_2_^−^, ^•^OH, and h^+^, respectively. For all samples, the additions of BQ result in the largest extent of photoactivity decrease, confirming ^•^O_2_^−^ be the main reactive species. Based above, 0.5CoPc/9P‐CN exhibits superior photocatalytic performance for visible‐light 2,4‐DCP degradation by more effectively activating O_2_, which is also found to possess favorable stability by examining the photocurrent densities and recycling experiments for five runs (Figure S9, Supporting Information).

## 3 Charge Transfer Mechanism and the Verification of Oxygen Species

**Figure 4:**
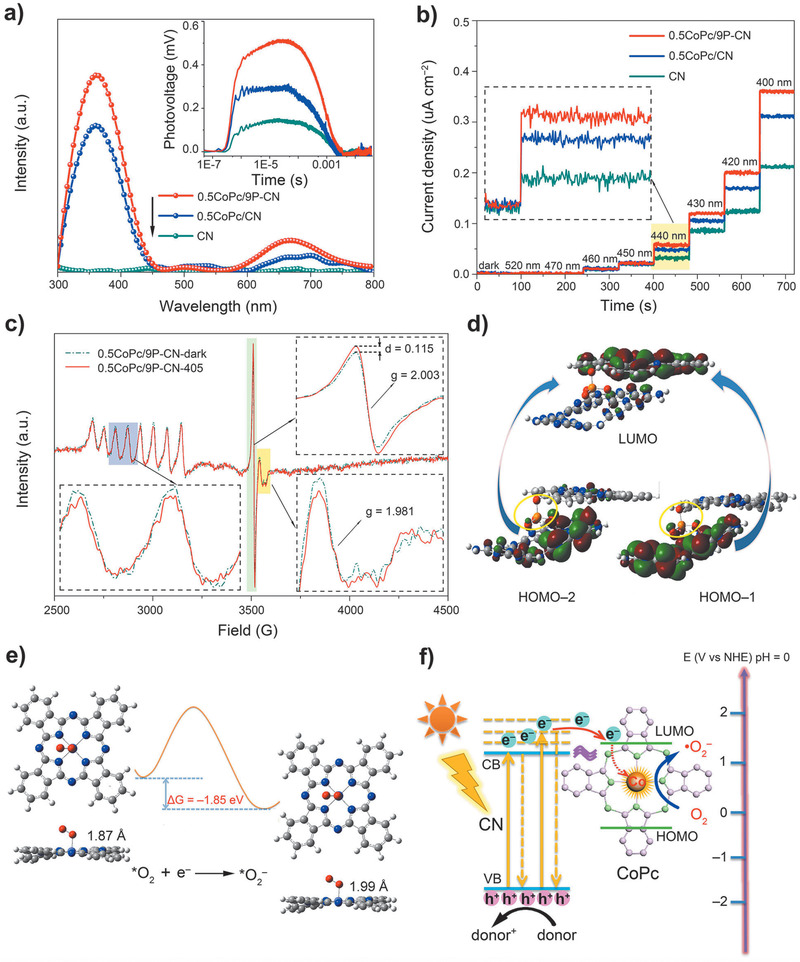
a) SPS responses in N_2_ atmosphere, TPV responses in air under the irradiation of 355 nm light (inset), and b) MPAS of CN, 0.5CoPc/CN, and 0.5CoPc/9P‐CN_,_ respectively. c) EPR spectra of 0.5CoPc/9P‐CN under the irradiation of 405 nm light. d) Contour surfaces of frontier molecular orbitals HOMO‐1, HOMO‐2, and LUMO for the excited state with the maximum oscillator strength. e) Free energy of *O_2_^−^ formation on single‐atom Co^2+^ of CoPc and comparison of Co—O bond length for *O_2_ and *O_2_^−^ adsorption on CoPc, respectively. f) Schematic of the main charge transfer mode and subsequent photoreduction process of O_2_ on the CoPc/P‐CN heterojunction under visible‐light irradiation.

In addition to the light absorption, the charge separation and subsequent catalytic process are the main factors determining the photoactivity. First, the charge separation properties of as‐fabricated nanocomposites were investigated by the steady‐state surface photovoltage spectroscopy (SPS) (**Figure** **4**a; Figures S10a and S11a, Supporting Information). No detectable SPS signal is observed for CN, while the resulting heterojunctions display obvious signals in the range of 300–450 nm due to the charge transport from CN to CoPc. The relatively weak signals among 550–800 nm are attributed to CoPc. As the CoPc amount increases, the signal becomes stronger than that of CN and reaches the maximum for 0.5CoPc/CN with the best charge separation. Whereas, further adding CoPc leads to an apparent signal decay due to the excessive aggregation. Besides, photoluminescence (PL) and fluorescence spectra (FS) related to formed hydroxyl radical amount reflect coincident results (Figure S10b,c, Supporting Information). It is suggested the interfacial charge transfer (ICT) do appear in the CoPc/CN heterojunctions. With the fixed amount of CoPc of 0.5%, the introduction of phosphates is found to further facilitate the charge separation till reaching an optimal amount for 0.5CoPc/9P‐CN (Figure S11a–c, Supporting Information). Over phosphates result in lower SPS signals, owing to the inhibition effect of aggregation on the charge transport. The transient‐state surface photovoltage (TPV) spectra under 355 nm laser were collected to study the dynamic processes of the photogenerated charge carriers. Figure 4a inset illustrates a consistent trend as SPS results. The time‐resolved PL (TR‐PL) spectra, photoelectrochemical linear sweep voltammetric (LSV) curves along with electrochemical impedance spectra (EIS) further support the SPS and TPV results (Figure S12, Supporting Information). Note that for all investigated samples, the photocatalytic performances for 2,4‐DCP degradation are found to be proportional to the charge separation (Figures S10d and S11d, Supporting Information).

Based on the results of diffuse reflection spectrum (DRS) (Figure S13a, Supporting Information) and the Mott–Schottky curve for CoPc (Figure S13b, Supporting Information), the LUMO level of CoPc is calculated to be −1.39 eV, which is slightly higher than the CB bottom level of 9P‐CN. Accordingly, the scanning Kelvin probe results imply the band edge position of CoPc might matches with 9P‐CN to enable the high‐level‐energy photoelectron transfer from CN to CoPc besides the normal direction of photoelectron transfer from CoPc to CN (Figure S13c, Supporting Information). Therefore, after verified enhanced charge separation, it is still necessary to intensively clarify the ICT mode. The monochromatic photocurrent action spectra (MPAS) were collected and illustrated in Figure 4b. Under dark and the excitation of light 520 and 470 nm, for three comparative samples negligible photocurrent densities are noticed, since both CN and CoPc are not excited. When CN instead of CoPc is excited by 460 and 450 nm light, two sets of identical photocurrent densities are demonstrated for three samples, indicating still no charge transfer appears in the heterojunctions. Notably, a bit sharp photocurrent densities for 440 nm are observed (Figure 4b inset), the photocurrent densities take on the sequence as CN < 0.5CoPc/CN < 0.5CoPc/P‐CN. This suggests that the light excitation at 440 nm begin to endow sufficient thermodynamic energy for the photogenerated electrons to realize the ICT from CN to CoPc, since the energy level of the LUMO of CoPc is slightly higher than the CB of CN. As the excitation wavelength further decreases from 440 to 400 nm with 10 nm as the step, the photocurrent densities of three samples dramatically increase, indicative of the greatly improved charge separation. Especially, the superiority of 0.5CoPc/9P‐CN on the charge separation becomes increasingly remarkable. Moreover, the ICT mode and the specific role of CoPc were further uncovered with the assistance of quasi‐in‐situ low‐temperature EPR. The characteristic EPR signals of CN locate at *g* = 2.003, originating from the unpaired electrons on conjugated CN aromatic rings (Figure S14b, Supporting Information).^[^ ^8^ ^]^ When CN is excited by the 405 nm light, the signal noted as CN‐405 is stronger than CN‐dark, which is due to more produced free electrons. For 0.5CoPc/CN, the observed signals at *g* = 2.003 are the overlay results of CN and CoPc. Since CoPc is not excited under 405 nm light, the contribution of CoPc to the signal of 0.5CoPc/CN is equal no matter under dark or 405 nm light. Hence, the difference value (*d* = 0.124) between the intensities of signals at *g* = 2.003 between 0.5CoPc/CN‐405 and 0.5CoPc/CN‐dark could semiquantify the free electrons produced by the excitation of CN for 0.5CoPc/CN (Figure S14d, Supporting Information). Similarly, the *d* value for 0.5CoPc/9P‐CN is measured to be 0.115 in Figure 4c upper right. By comparing two *d* values, it could be concluded that the introduction of phosphates facilitates more photogenerated electrons transfer from CN to CoPc under identical conditions (405 nm). This finding is well consistent with the SPS, TPV, and MPAS results.

As for the probed ICT mode from CN to CoPc, the hypothesis that the photogenerated electrons would transfer specifically from CN to the ligand of CoPc then to the expected Co–N_4_ (II) sites needs to be further validated. For pure CoPc, the EPR signals of CoPc‐dark and CoPc‐405 totally overlap, centering at *g* = 1.981 and typically assigned to Co^2+^ (Figure S14c, Supporting Information).^[^ ^31^ ^]^ In each spectrum of 0.5CoPc/CN‐dark, 0.5CoPc/CN‐405, 0.5CoPc/9P‐CN‐dark, and 0.5CoPc/9P‐CN‐405 (Figure S14a, Supporting Information; Figure 4c left), besides the identical signal for Co^2+^ as pure CoPc, an axial signal with an eight‐line splitting pattern is observed, resulting from the hyperfine coupling of the unpaired electron of Co^2+^.^[^ ^31^, ^32^ ^]^ Notably, it is undetectable for CoPc solid due to the intermolecular aggregation.^[^ ^33^ ^]^ The characteristic signal for Co^2+^ decreases when 0.5CoPc/9P‐CN is excited by the 405 nm light, indicating that the photoelectrons from CN could specifically transfer to the Co^2+^ centers as partially reduced. Similar Co^2+^ signal changes could be also observed for 0.5CoPc/CN (Figure S14d, Supporting Information). Therefore, the ICT mode could be convincingly confirmed that when CN is excited with proper wavelength, the photogenerated electrons would transfer to the ligand of CoPc then to the single Co–N_4_ (II) sites.

Another matter could not be ignored is the sensitization effect of CoPc in the CoPc‐involved heterojunctions. When only CoPc is excited by 660 nm light, it is inferred the photogenerated electrons would transfer from CoPc to CN. As expected, no detectable changes of Co^2+^ signals could be observed for 0.5CoPc/CN‐660 or 0.5CoPc/9P‐CN‐660, compared with the corresponding samples under dark (Figure S14e,f, Supporting Information). In addition, the monochromatic FS spectra related to the produced hydroxyl radicals were collected as supplementary (Figure S15, Supporting Information). Normally the amounts of photoinduced hydroxyl radicals are attributed to be proportional to the charge separation. The intensities of FS signals increase as the order CN‐405 < 0.5CoPc/CN‐405 < 0.5CoPc/9P‐CN‐405, consistent with the EPR results under the irradiation of light 405 nm. Interestingly, when CoPc is excited by 660 nm light, CN‐660 shows negligible signal meanwhile 0.5CoPc/CN‐660 and 5CoPc/9P‐CN‐660 demonstrate obvious signals, especially for the latter, implying the existence of the ICT mode from CoPc to CN. Moreover, the single‐wavelength photoactivities of 0.5CoPc/9P‐CN for the 2,4‐DCP degradation were examined under 405 and 660 nm light, respectively (Figure S16, Supporting Information). It is further confirmed that both ICT modes simultaneously exist, however the mode that from CN to CoPc then to single‐atom Co^2+^ centers makes the major contribution. Noteworthily, time‐dependent density functional theory (TDDFT) calculation was carried out for our model structure (CoPc/P‐CN) in order to acquire the charge transfer characteristic. The calculated results were shown in Table S1 in the Supporting Information. The maximum absorption with strongest oscillator strength comes from the transition of S0→S13. The excited state S13 could be described as a linear addition of main configurations HOMO‐2→LUMO and HOMO‐1→LUMO. Figure 4d gives the contour surfaces of the frontier orbitals HOMO‐2, HOMO‐1, and LUMO relevant to the maximal absorption. Obviously, both transitions of HOMO‐2→LUMO and HOMO‐1→LUMO shows ICT characteristic, that is the charge transfer from CN to CoPc, which is consistent with above experimental results.

Based on the evidenced ICT mode, the photogenerated electrons transferred to the single Co–N_4_ (II) sites would induce subsequent reduction reactions. As expected, 0.5H_2_Pc/CN exhibits a similar onset potential compared with CN, which is much lower for CoPc‐modified ones as expected. It is indicated that CoPc is more favorable for O_2_ reduction owing to the catalytic effect of Co^2+^ (Figure S17, Supporting Information). In particular, it is the largest onset potential for 0.5CoPc/9P‐CN. In addition, the temperature‐programmed desorption (TPD) curves of O_2_ further demonstrate 0.5CoPc/9P‐CN displays the best affinity with O_2_ (Figure S18, Supporting Information). Interestingly, the activation and photoreduction of O_2_ on CoPc were investigated by the theoretical calculation. As shown in Figure 4e, O_2_ could adsorb on CoPc stably with Co—O bond (1.87 Å). Subsequently, *O_2_^−^ would be formed immediately after activating O_2_ with the photoelectrons owing to the lower free energy (−1.85 eV) and longer Co—O bond length (1.99 Å). When producing *O as another possible active oxygen species, the lowered free energy is −1.67 eV (Figure S19, Supporting Information). This indicates a preferable production of ^•^O_2_^−^ for the activation of O_2_. Summarily, the photoelectrons would transfer from CN along the phosphates to the ligand of CoPc and then to the single Co^2+^ sites, and subsequently induce the reduction of O_2_ under visible‐light irradiation, finally resulting in the main ^•^O_2_^−^ radicals to dominate the oxidative 2,4‐DCP degradation on CoPc/P‐CN heterojunctions (Figure 4f).

In supplementary, the degradation process mechanism of 2,4‐DCP is further investigated based on the identified intermediates (Figure S20, Supporting Information) by liquid chromatography tandem mass spectrometry (LC‐MS), as proposed to be a two‐step dechloration consequently to the final mineralization (Figure S21, Supporting Information).

## 4 Applicability and Outlook

**Figure 5:**
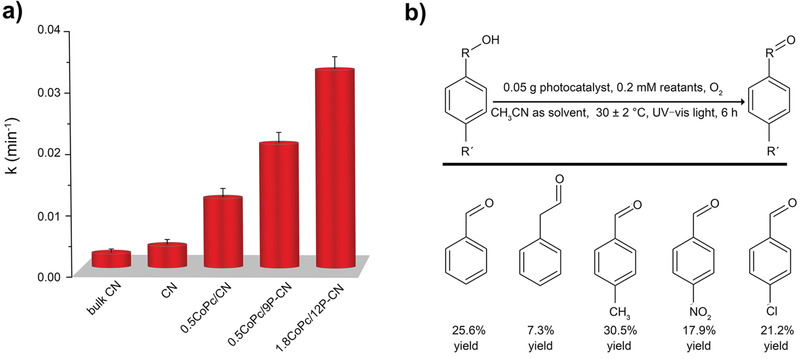
Photoactivity potential evaluation for a) 2,4‐DCP degradation and b) selective oxidation of aromatic alcohols using 1.8CoPc/12P‐CN with O_2_ as the oxidant under UV–vis light irradiation. Data are presented as the mean ± SD from three independent experiments.

The strategy of phosphate modulated CoPc/CN heterojunctions is validated to be effective based on above designed experiments. Considering the probed ICT mode, it is natural to understand a determined amount of phosphates containing hydroxyl groups has an upper limit for forming H‐bonds to assist the high dispersion of ultrathin CoPc assemblies then to favor the photocatalytic reaction, since excess phosphates might aggregate on CN surface to inhibit the charge transfer. Moreover, excess CoPc would certainly thicken the assemblies hence retard the charge separation. Thus, by mutually modulating the amounts of phosphates and CoPc, the optimum 1.8CoPc/12P‐CN sample has been obtained to reach the largest loading amount of CoPc with proper thickness and enriched single Co^2+^ sites (Figure S22, Supporting Information). Under the UV–vis light irradiation, this optimized sample exhibits an impressive photoactivity improvement compared with 0.5CoPc/9P‐CN (**Figure** **5**a). Notably, the degradation rate of 1.8CoPc/12P‐CN reaches about 14‐time of that for bulk CN, demonstrating the superiority of as‐designed CoPc/P‐CN heterojunctions. Considering the probed capability of activating O_2_ effectively, resultant CoPc/P‐CN heterojunctions could potentially be expanded to other oxidative transformations. The optimized 1.8CoPc/12P‐CN has also been applied for the selective oxidation of various aromatic alcohols as shown in Figure 5b. Under the atmospheric pressure, the oxidation of benzyl alcohol as a typical substrate has obtained a remarkably high yield of 25.6% to produce the benzyl aldehyde as the main product. As the scavenger experiments demonstrated, the ^•^O_2_^−^ radicals predominantly affect the photoactivity (Figure S23, Supporting Information). Even for the substrates with the withdrawing groups like –NO_2_ or –Cl, which are relatively difficult to be converted, favorable photocatalytic performances were also obtained. By contrast with reported MPc/semiconductor and CN‐based photocatalysts, 1.8CoPc/12P‐CN has obviously exhibited significant advantages in terms of as‐uncovered mechanism insight as well as the photocatalytic performance (Tables S2 and S3, Supporting Information).

## 5 Conclusion

In summary, a facile strategy of phosphate‐induced highly dispersed ultrathin CoPc assemblies on CN nanosheets via the strengthened H‐bonding effect has been successfully developed to construct efficient CoPc/P‐CN heterojunctions based on theoretical guidance. The optimized heterojunction exhibits exceptional promotion for O_2_ activation, leading to the excellent photoactivities for the aerobic degradation of 2,4‐DCP and oxidation of aromatic alcohols. Especially, the ICT and subsequent catalytic mechanism via abundant single Co–N_4_ (II) sites for O_2_ activation to form ^•^O_2_^−^ as the main reactive specie is for the first time uncovered in detail for MPc‐based heterogeneous photocatalysts, assisted by the theoretic calculation and designed experiments. This work exploits the potential of MPc in the photocatalytic O_2_ activation. More importantly, we provide an easily scale‐up strategy to fabricate highly dispersed ultrathin MPc‐based heterojunctions containing abundant single‐atom metal sites as excellent photocatalysts by modulating the interfacial H‐bonding interaction.

## Conflict of Interest

The authors declare no conflict of interest.

## Supporting information

Supporting Information
