# Supplementary material for: Ultrathin Phosphate‐Modulated Co Phthalocyanine/g‐C3N4 Heterojunction Photocatalysts with Single Co–N4 (II) Sites for Efficient O2 Activation
Source: Adv Sci (Weinh). 2020 Jun 25;7(16):2001543. doi: 10.1002/advs.202001543 (PMC7435235; doi:10.1002/advs.202001543)
Supplement: Supplementary file 1 — Supporting Information [file ADVS-7-2001543-s001.pdf]

WILEY-VCH

Copyright WILEY-VCH Verlag GmbH & Co. KGaA, 69469 Weinheim, Germany, 2020.

Supporting Information

Ultrathin phosphate-modulated Co phthalocyanine/g-C_3N_4 heterojunction photocatalysts with single Co-N_4 sites for efficient O_2 activation

Xiaoyu Chu, Yang Qu, Amir Zada, Linlu Bai, Zhijun Li, Fan Yang, Lina Zhao, Guiling Zhang, Xiaojun Sun, Zhao-Di Yang,* and Liqiang Jing**

1. Computation Section

1.1 Computation details

The equilibrium geometrical optimizations were completed using DFT/WB97XD method implemented in the Gaussian 09 package.[1,2] Gradient optimizations were carried out using the 6-31G* basis set for C, O, P, N, and H and LANL2DZ for metal atom(Co, Fe, Ni, Cu, Zn). The stationary frequency calculations at 298.15 K and 1 atm were performed at the same level for each of the optimized structures to examine any imaginary frequency or the corresponding vibrational modes. TDDFT (time-dependent density functional theory) was employed to predict excited state energies and properties to obtain the information of charge transfer.[1]

For giving more reliable interaction energies (Adsorption Energies, $E_{ad,}$ or Bonding energies, E_b), the Basis Set Superposition Error (BSSE) corrections are carried out. E_{ad} or E_b can be obtained according to the formula E_{ad}/E_b = E_corrected-(E(1) + E(2) +... E(j) ... +E(n)), in which E_corrected = E_complex + E_BSSE and E(j) means the energy doing monomer centered basis set (MCBS) calculation for fragment j.

2. Experimental Section

2.1 Chemicals

All phthalocyanines CoPc (II), FePc (II), CuPc (II), ZnPc (II), NiPc (II) and H_2Pc were purchased from Sigma-Aldrich and other chemicals (analytical grade) were purchased from Aladdin. All reagents were directly used without further purification. Deionized water was used throughout.

2.2 Material synthesis

Synthesis of bulk CN**:** The bulk CN was simply obtained by the calcination of melamine.[3] In a typical experiment, a proper amount of melamine was taken in the ceramic crucible and heated in the muffle furnace at 500 °C for 4 h at a temperature ramp of 5 °C min^{-1}.

Synthesis of ultrathin CN nanosheets**:** Firstly, melamine (10 g) and cyanuric acid (4 g) were dispersed in (500 mL) deionized water. The mixed suspension of cyanuric acid and melamine was stirred for 3 h at 80 °C, then cooled to room temperature, centrifuged at 4000 rpm for 15 min, washed with deionized water and ethanol several times, then dried overnight at 80 °C. The obtained dried powder was transferred into a closed quartz boat and heated from room temperature to 550 °C with a heating rate of 5 °C min^{-1} under air atmosphere for 4 h, which product then undergone a second-time calcination treatment in the semi-closed porcelain boat at 500 °C for 2 h under air atmosphere. After natural cooling to room temperature, as-obtained fluffy product was treated by HNO_3 solution then dried overnight at 80 °C to obtain final CN nanosheets.

Synthesis of CoPc/CN nanocomposites**:** CN nanosheets (1 g) were dispersed in ethanol of (30 mL) under ultrasonication for 30 min. Following, a calculated volume of CoPc solution with ethanol as solvent was added into the CN suspension followed by the sonication and stirring for 30 min to obtain a well-mixed suspension. The suspension was further stirred at room temperature for 24 h then get the solvent completely evaporated under vigorous stirring at 80 °C. As-obtained solid was dried overnight at 80 °C and then calcined at 250 °C for 1 h in a muffle furnace with a heating rate of 5 °C min^{-1}. These samples were represented by XCoPc/CN, where X indicates the mass ratio (0.05, 0.1, 0.5 and 1%) of CoPc to CN.

***Synthesis of phosphate-modified CN*:** CN nanosheets (1 g) were added into the NaH_2PO_4 solution (100 mL) with desired concentration. The mixed suspension was treated under ultrasonication for 30 min then dried at 80 °C and then annealed at 450 °C for 1 h. The obtained powders were denoted as YP-CN, where Y indicates the the mass ratio (3, 6, 9 and 12%) of NaH_2PO_4 to CN.

***Synthesis of ultrathin phosphate-modulated CoPc/CN*:** Same procedures were performed as the synthesis of CoPc/CN by replacing CN to P-CN to obtain XCoPc/YP-CN nanocomposites.

2.3 Sample characterization

The X-ray powder diffraction (XRD) patterns of the samples were measured with a Bruker D8 Advance diffractometer, using Cu Ka radiation. Thermal gravimetric (TG, Netzsch, STA 2500 Regulus) analysis under air flow were conducted for the biomass carbon, PW and PCM composites at a heating ramp of 10 °C min^{-1}. The Brunauer-Emmett-Teller (BET) surface area was measured with an ASAP2020Plus 1.03 apparatus (Micromeritics Instrument Corp.). The UV–vis diffuse reflectance spectra (UV–vis DRS) and the UV–vis absorption spectra of the samples were measured with a Model Shimadzu UV2550 spectrophotometer, using $BaSO_4$ as a reference. The Fourier-transform infrared (FT-IR) spectra of the samples were collected with a Bruker Equinox 55 Spectrometer, using KBr as diluents. The FT-IR spectra in the figures have been normalized to the background before and after the main features. The photoluminescence (PL) spectra of the samples were measured with a spectrofluoro-photometer (LS55 Perkin-Elmer) at excitation wavelength of 300 nm. The X-ray photoelectron spectroscopy (XPS) technique was used to detect the surface composition and elemental chemical state of the samples, by using a Model VG ESCALAB apparatus with Mg K X-ray source, and the binding energies were calibrated with respect to the signal for adventitious carbon (binding energy = 284.6 eV). The morphology of the samples was

analyzed by transmission electron microscopy (TEM) on a FEI Tecnai G2 S-Tw in instrument with an acceleration voltage of 200 kV. The FEI Tecnai G2 S-Tw in equipped with Energy dispersive X-ray (EDX) Detector was used to acquire element analyses of the samples. The surface photovoltage spectroscopy (SPS) measurements for samples were implemented on home-built apparatus, equipped with a lock-in amplif ier (SR830, USA) synchronized w ith a light chopper (SR540, USA). The transient-state surface photovoltage (TPV) measurement was performed in air atmosphere and at room temperature. The samples were excited by a radiation pulse of 355 nm with 10 ns width from a second harmonic Nd: YAG laser (Lab-130-10H, Newport, Co.). The signal was amplified with a preamplifier and then registered by a 1 GHz digital phosphor oscilloscope (DPO4104B, Tektronix). Time-resolved PL (TR-PL) spectra were recorded with a single photon counting spectrometer from (Edinburgh Instrument, FLS 920) with 1μs pulse lamp as the excitation. Scanning Kelvin probe (SKP) measurements (SKP5050 system, Scotland) were carried out at room temperature. The thickness of the samples was analyzed by Atomic force microscopy (AFM) on a multimode nanoscope VIII instrument (Bruker) with mica as the base. The Raman spectra were recorded with a Renishaw in Via Confocal Raman spectrometer with a 785 nm laser as excitation source. The electron paramagnetic resonance (EPR) measurements were carried out on a Bruker EMX plus model spectrometer operating at the X-band frequency.

2.4 Analysis of hydroxyl radicals

The sample (0.05 g) was dispersed in (50 mL) coumarin aqueous solution (0.001 mol L^{-1}) in a beaker. Prior to irradiation, the reactor was magnetically stirred for 10 min to attain an adsorption-desorption equilibrium in dark. After irradiation for 1 h with a 150 W Xenon lamp (GYZ220 made in China), the sample was centrifuged, and then a certain volume of solution was transferred into a Pyrex glass cell for the fluorescence measurement of 7-

hydroxycoumarin at 332 nm excitation wavelength with an emission peak wavelength at 460 nm through the above mentioned spectrofluorometer.

2.5 Evaluation for O_2 temperature-programmed desorption (O_2-TPD)

The O_2-TPD was measured with a home-built flow apparatus. The typical method is as follows: the sample (50 mg) was pre-heated to 300 °C maintaining for 0.5 h to remove any moisture and then cooled to room temperature under an ultra-high-pure He stream with a flow rate of 30 mL min^{-1}. After that, the system was cooled to room temperature and then the sample was blown continuously with O_2 for 60 min at 30 °C. The excess weakly adsorbed O_2 was removed by exposure to ultra-high pure He. Then the temperature was increased to 500 °C with a heating rate of 10 °C min^{-1} under pure He.

2.6 Photoelectrochemical (PEC) and electrochemical (EC) experiments

Photoelectrochemical (PEC) and electrochemical (EC) reduction measurements were carried out in a traditional three-electrode system. The prepared sample was used as working electrode, a platinum plate (99.9%) was used as the counter electrode, a saturated KCl Ag/AgCl electrode was used as the reference electrode, and (0.5 mol L^{-1}) Na_2SO_4 solution as the electrolyte. High purity nitrogen gas (99.999%) was bubbled through the electrolyte before and during the experiments. PEC experiments were performed in a quartz cell using a 500 W xenon lamp with a cut-off filter (λ > 420 nm) as the illumination source. An IVIUM V13806 electrochemical workstation was employed to test photoelectrochemical and electrochemical performance of a series of catalysts. All the experiments were performed at room temperature (about 25 °C).

2.7 EPR measurements

The $•O_2^−$ radicals usually can be trapped by 5,5-dimethyl-l-pyrroline N-oxide (DMPO), producing the EPR signals of their adducts. The sample (5 mg) was dissolved in DMPO solution with CH_3OH as solvent to obtain the liquid mixture. After illumination, the mixture was characterized using a Bruker EMX plus model spectrometer operating at room temperature.

2.8 Photocatalytic performance measurements

Photocatalytic degradation of organic contaminants: The photocatalytic activities of the as-prepared samples were evaluated by degrading 2,4-DCP as model pollutants. A 150 W Xenon lamp was used as a light source. The photocatalyst (0.1 g) was dispersed in the aqueous 2,4-DCP solution (20 mg $L^{−1}$, 50 mL). Prior to irradiation, the suspension was magnetically stirred in dark for 30 min to ensure the establishment of an adsorption/desorption equilibrium. The aqueous solutions were fully mixed by stirring for 1 h in an open glass reactor under visible-light irradiation and the concentration of 2,4-DCP was analyzed by the UV spectrometer. The intermediates were analyzed with Agilent liquid chromatography tandem mass spectrometry (LC-MS, 6410MS, USA). The intermediate fragments of the main reaction were analyzed through scan mode.

Recycle test: After each run for 3 h, the used photocatalyst was separated, washed with deionized water and acetone then dried at 80 ^{o}C in the vacuum oven for the next cycle. The concentration of 2,4-DCP in the liquid reaction mixture was measured by the UV spectrometer.

Photocatalytic alcohol oxidation: The photocatalyst (50 mg) and the alcohol as reactant (200 μmol) were added to toluene as solvent (5 mL) within a single flask (25 mL), which was sealed with rubber septum caps. The photocatalyst was dispersed well by ultrasonication for 5 min, and O_2 was bubbled through the solution for 20 min. The flask with the reflux of cooling water was immersed in a temperature-controlled water bath (30 ± 2 ^oC) under stirring (600 rpm) and irradiated under full light using a 300 W Xe lamp. Dodecane was applied as the external standard. The liquid mixture was analyzed using an Agilent gas chromatograph 6890 equipped with a HP-5 capillary column (30 m long and 0.32 mm in diameter, packed with silica-based supel cosil) and flame ionization detector (FID). The injector temperature was 250 °C and the spilt is (0.1 μL). The column head pressure of the carrier gas (helium) during the analysis was maintained at 22.57 psi. Temperature program: 50 °C to 180 °C; 20 °C min^{-1}; hold for 4 min. The by-products were identified by a gas chromatography-mass spectrometry (GC-MS, Agilent, GC 6890N, MS 5973 inert).

The active species trapping experiment: Tiny amount of scavenger including disodium ethylenediaminetetraacetate (EDTA), triethanolamine (TEA), 1,4-benzoquinone (BQ), isopropyl alcohol (IPA) was added in the photocatalytic system, respectively, to find out the effects of the corresponding active species on the photocatalytic reaction. Additionally, the EDTA (for aquaous system) and TEA (for organic system) are applied as h^+ scavenger, BQ as $\bullet O_2^-$ radical scavenger and IPA as •OH radical scavenger, respectively.

2.9 Statistical analysis

All the data were presented as means ± standard deviation (SD). In order to test the significance of the observed differences between the study groups, analysis by variance (ANOVA) statistics was applied and a value of $P < 0.05$ was considered to be statistically significant.

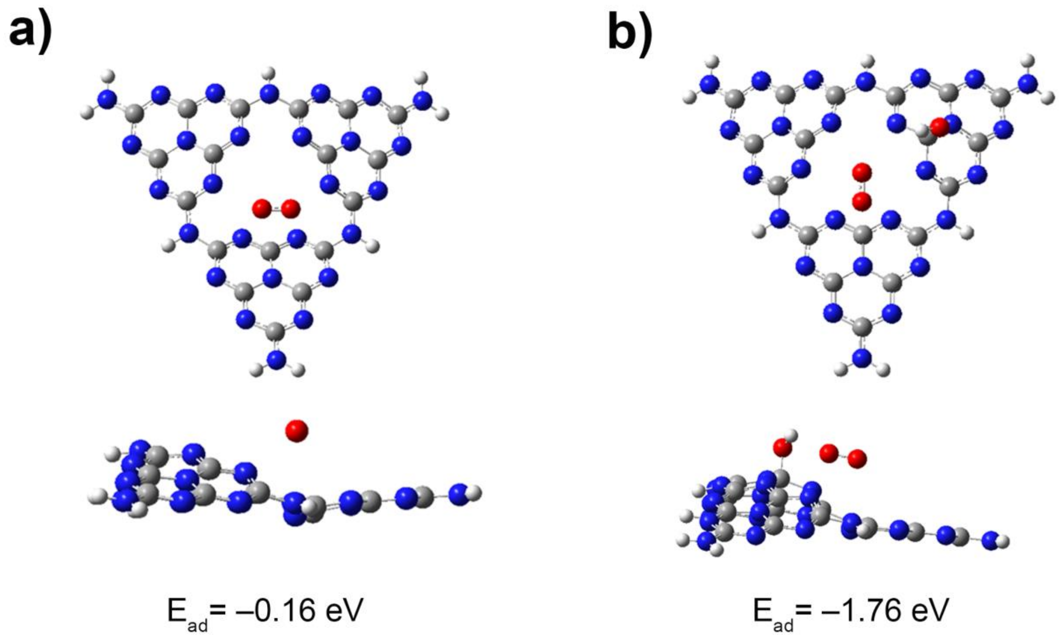

Figure S1. O_2 adsorption energies on pristine CN and hydroxylated CN.

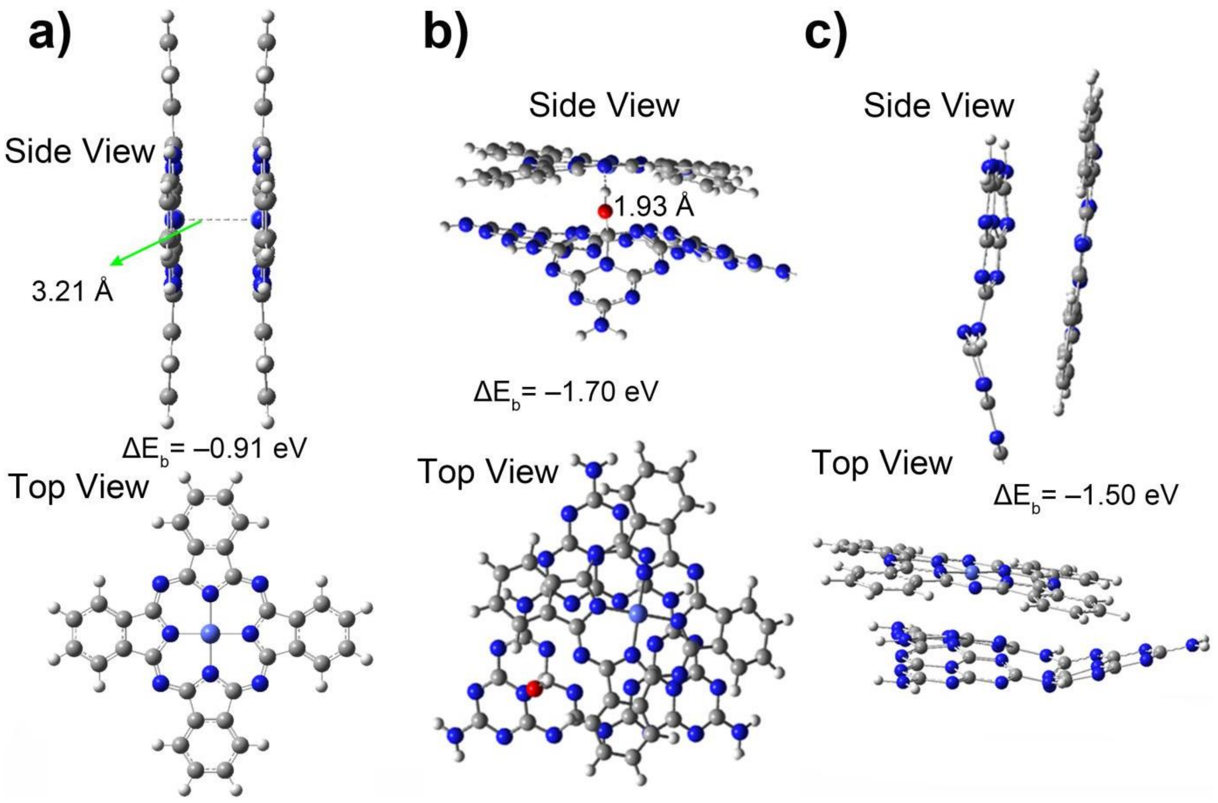

Figure S2. a) ΔE_b of the π–π interation between two CoPc molecules. b) ΔE_b of the H-bonding interaction between CoPc and hydroxyl-CN. c) ΔE_b of the π–π interation between CoPc and CN.

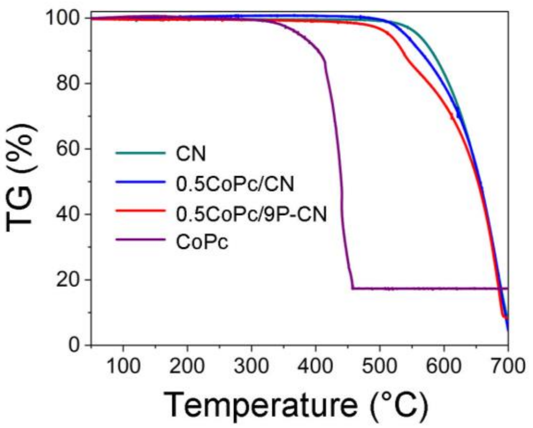

Figure S3. The TG curves for CoPc, CN, 0.5CoPc/CN and 0.5CoPc/9P-CN, respectively.

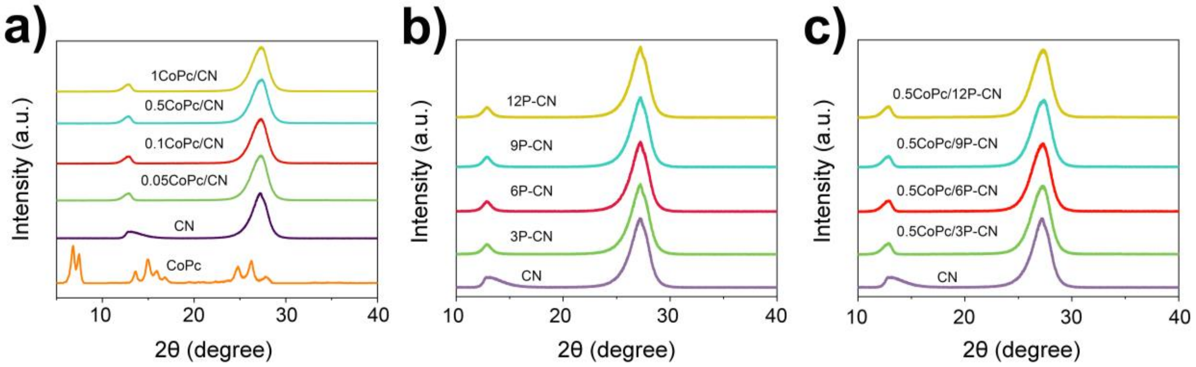

Figure S4. XRD patterns of a) CN and XCoPc/CN, b) CN and YP-CN and c) CN and 0.5CoPc/YP-CN.

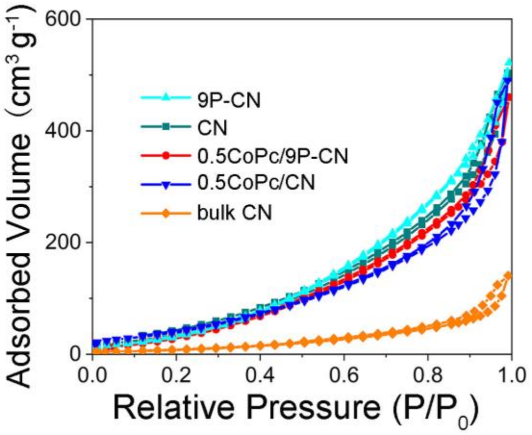

Figure S5. N_2 adsorption isotherms of bulk CN, CN, 9P-CN, CoPc/CN and 0.5CoPc/9P-CN, respectively.

The specific surface areas of bulk CN and CN are 35 and 207 m^2 g^{-1}, respectively, indicating the greatly enlarged surface area of CN by the two-dimensional morphology. Subsequently, for 9P-CN it's observed that the modification of phosphates leads to a slightly higher surface area of 224 m^2 g^{-1}. Furthermore, after loading CoPc on CN or 9P-CN, the surface areas of CoPc/CN and 0.5CoPc/9P-CN are 187 and 216 m^2 g^{-1}, respectively, exhibiting slightly decrease compared corresponding supports.

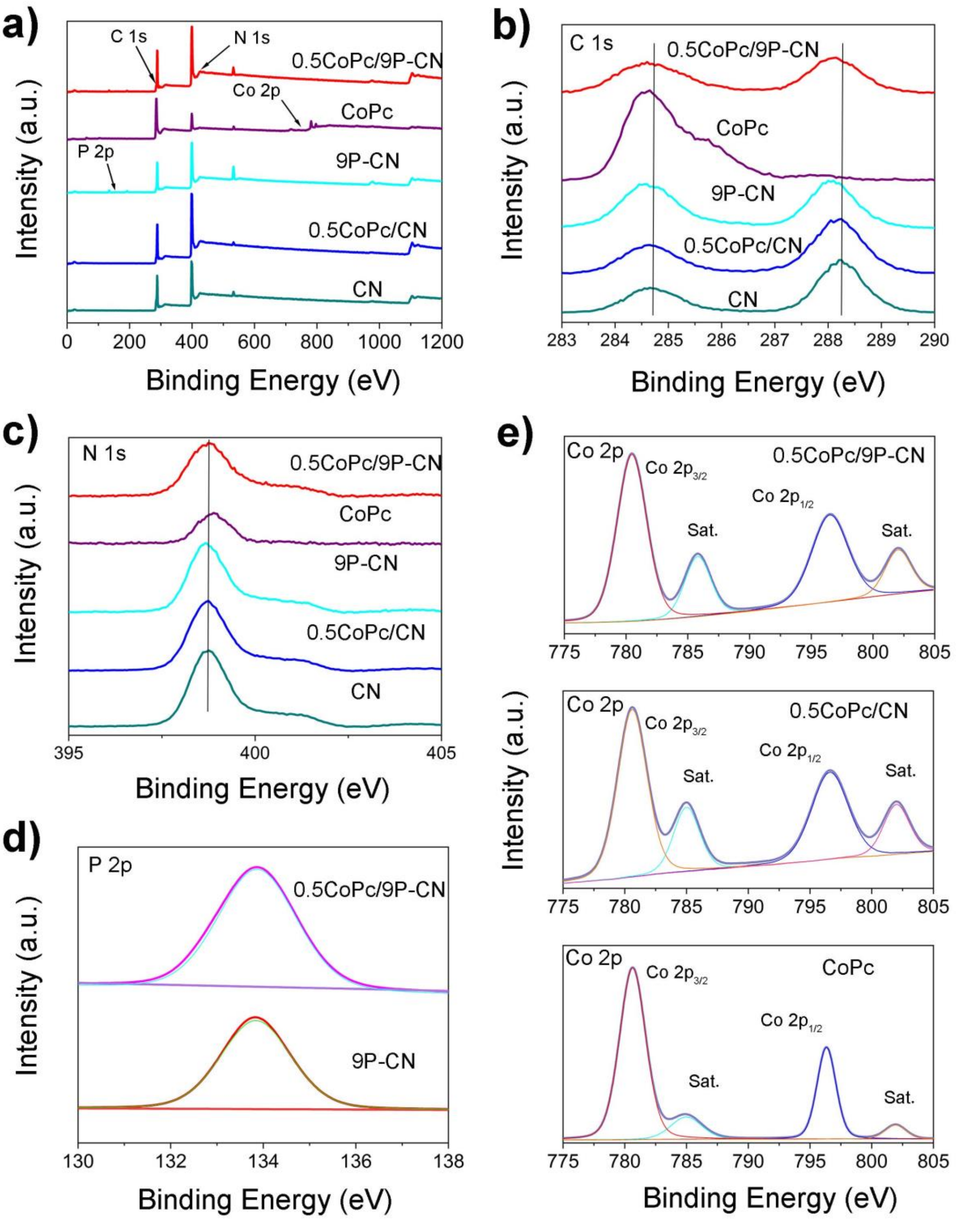

Figure S6. a) XPS survey spectra of CN, CoPc, 9P-CN, 0.5CoPc/CN and 0.5CoPc/9P-CN, respectively. b) The high-resolution XPS spectra of the C 1s region and c) the N 1s region for CN, CoPc, 0.5CoPc/CN, 9P-CN, 0.5CoPc/9P-CN, respectively. d) The high-resolution XPS spectra of the P 2p region for 9P-CN and 0.5CoPc/9P-CN, respectively. e) The high resolution XPS spectra of the Co 2p region for CoPc, 0.5CoPc/CN and 0.5CoPc/9P-CN, respectively.

The XPS survey spectra of CN, CoPc, 9P-CN, 0.5CoPc/CN and 0.5CoPc/9P-CN were displayed in Figure S6a. For CN, the XPS spectra of C 1s exhibit two main peaks at 288.0 and

284.6 eV, respectively, as normally assigned to N–C=N and C–C (Figure S6b).[4] By contrast, both the C 1s peaks for 9P-CN appear shift to lower binding energies, while the N 1s peaks show no obvious change compared with those for CN. This indicates the phosphate modification mainly affect carbon in CN, which is consistent with the theoretical model for P-CN since the hydroxyl groups locate on the C atoms. Furthermore, the loading of CoPc does not obviously change the C 1s and N 1s peaks of CN or 9P-CN, which reflects that no strong covalent interaction appear between CoPc and support. In our work, Raman and FT-IR along with DFT calculations have validated the interaction between CoPc with support is H-bonding as described in the main text. The Co 2p spectra for pristine CoPc, 0.5CoPc/CN and 0.5CoPc/9P-CN have been refined and illustrated, respectively, in Figure S6e. For pristine CoPc, two Co2p peaks at 795.6 eV and 780.6 eV along with the satellite peaks are attributed to Co $2p_{1/2}$ and Co $2p_{3/2}$ transitions, respectively. Compared with pristine CoPc, the intensities of Co 2p peaks for 0.5CoPc/CN and 0.5CoPc/9P-CN obviously decrease, which is due to the tiny CoPc as-loaded on CN and P-CN. Moreover, negligible Co 2p peak shifts could be observed, indicating the interfacial interaction between CoPc and CN or P-CN did not significantly affect the coordination environment of cobalt atoms. This is in agreement with the H-bonding-dominated model for CoPc/CN and CoPc/P-CN.

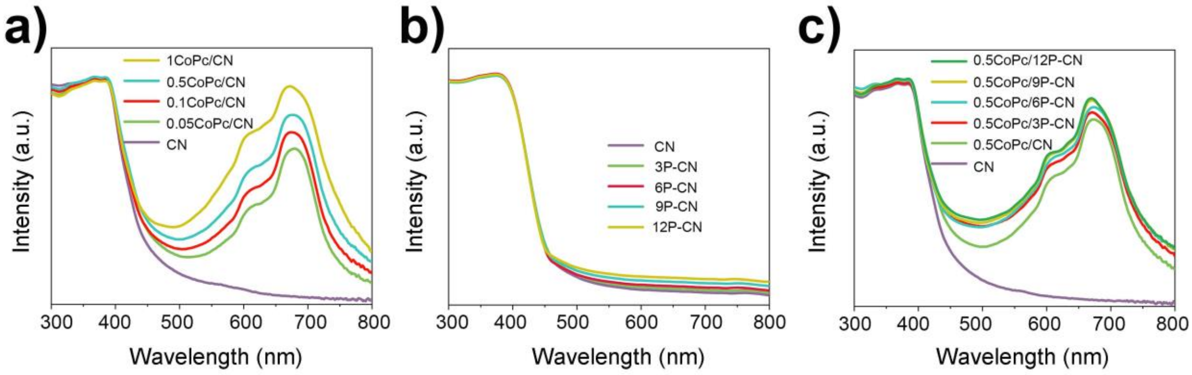

Figure S7. UV–vis absorption spectra of a) CN and XCoPc/CN, b) of CN and YP-CN, c) CN and 0.5CoPc/YP-CN.

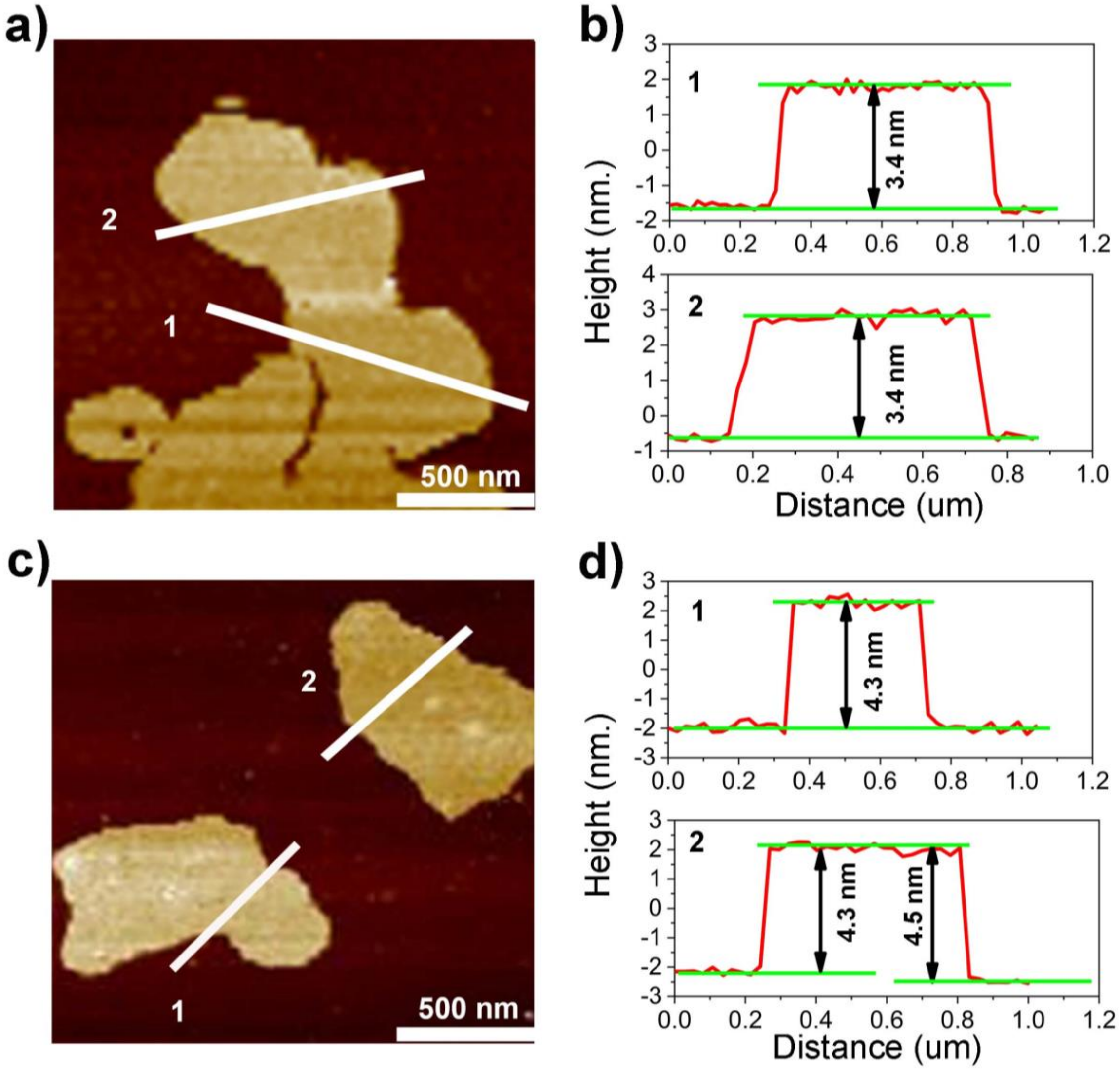

Figure S8. a) The AFM images of CN and b) the corresponding height profiles of CN. c) The AFM images and d) the corresponding height profiles of 0.5CoPc/CN.

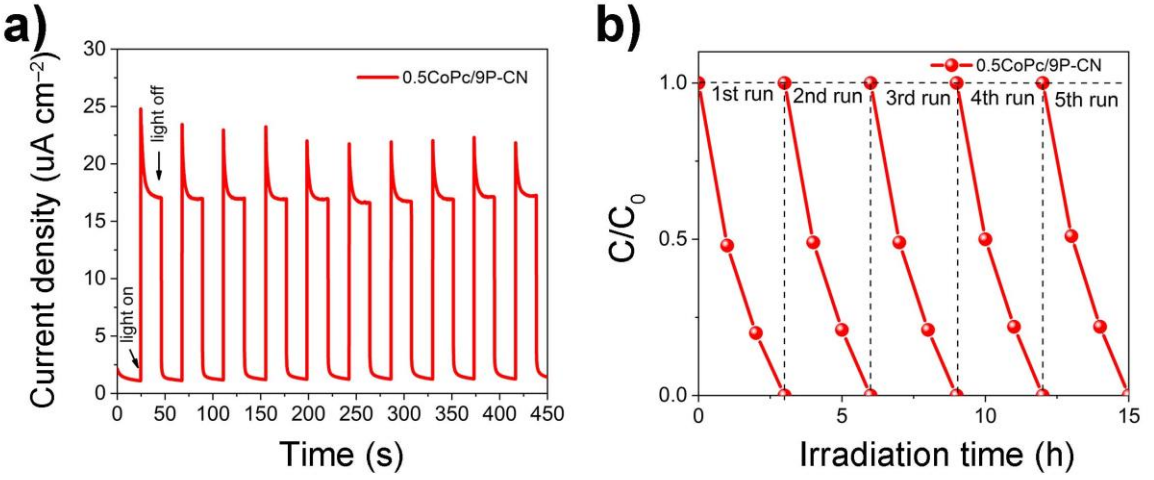

Figure S9. a) Electrochemical stability of 0.5CoPc/9P-CN. b) Photocatalytic cycling tests with 0.5CoPc/9P-CN under visible-light irradiation for the degradation of 2,4-DCP.

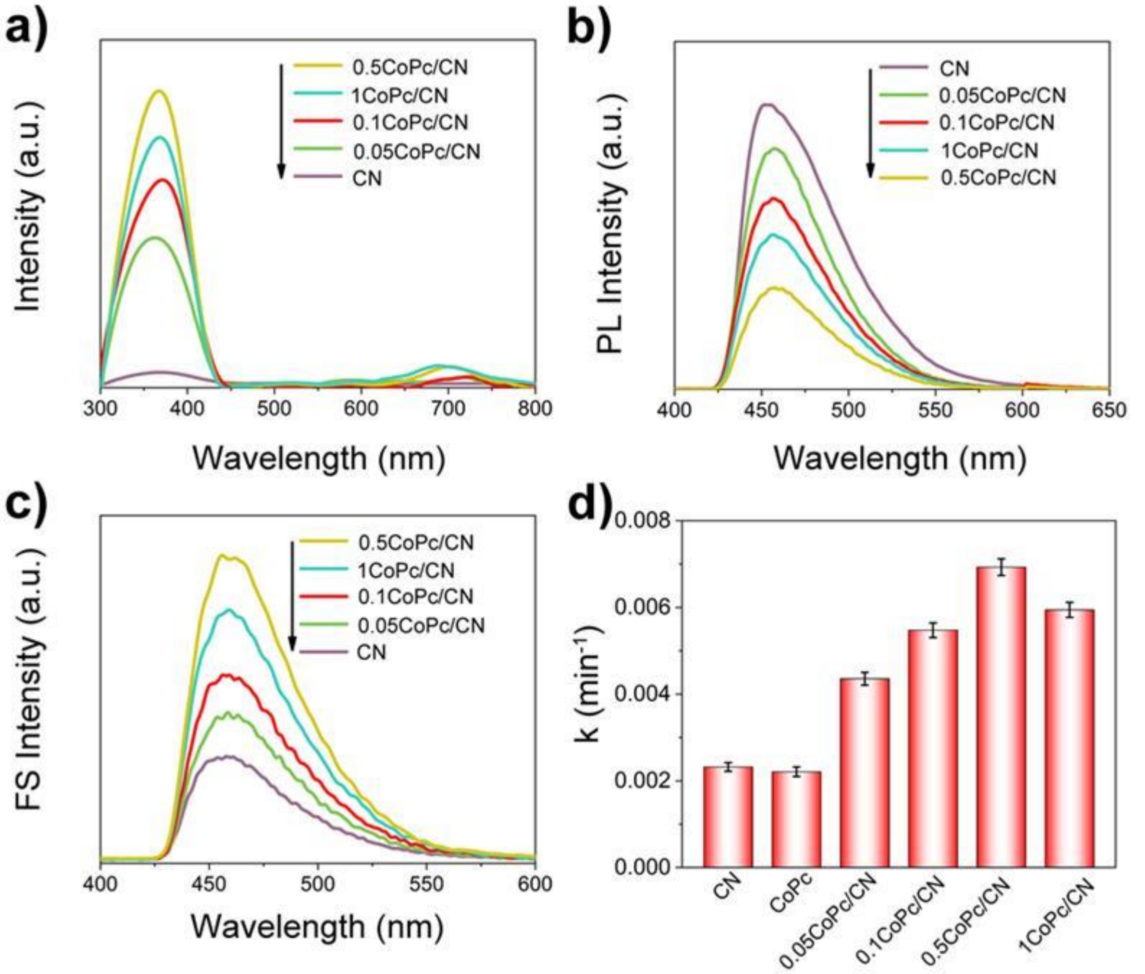

Figure S10. a) SPS spectra, b) PL spectra and c) fluorescence spectra related to the formed hydroxyl radicals under visible-light irradiation of CN and XCoPc/CN. d) Photocatalytic activities for 2,4-DCP degradation of CN and XCoPc/CN under visible-light irradiation. Data are presented as the mean ± SD from three independent experiments.

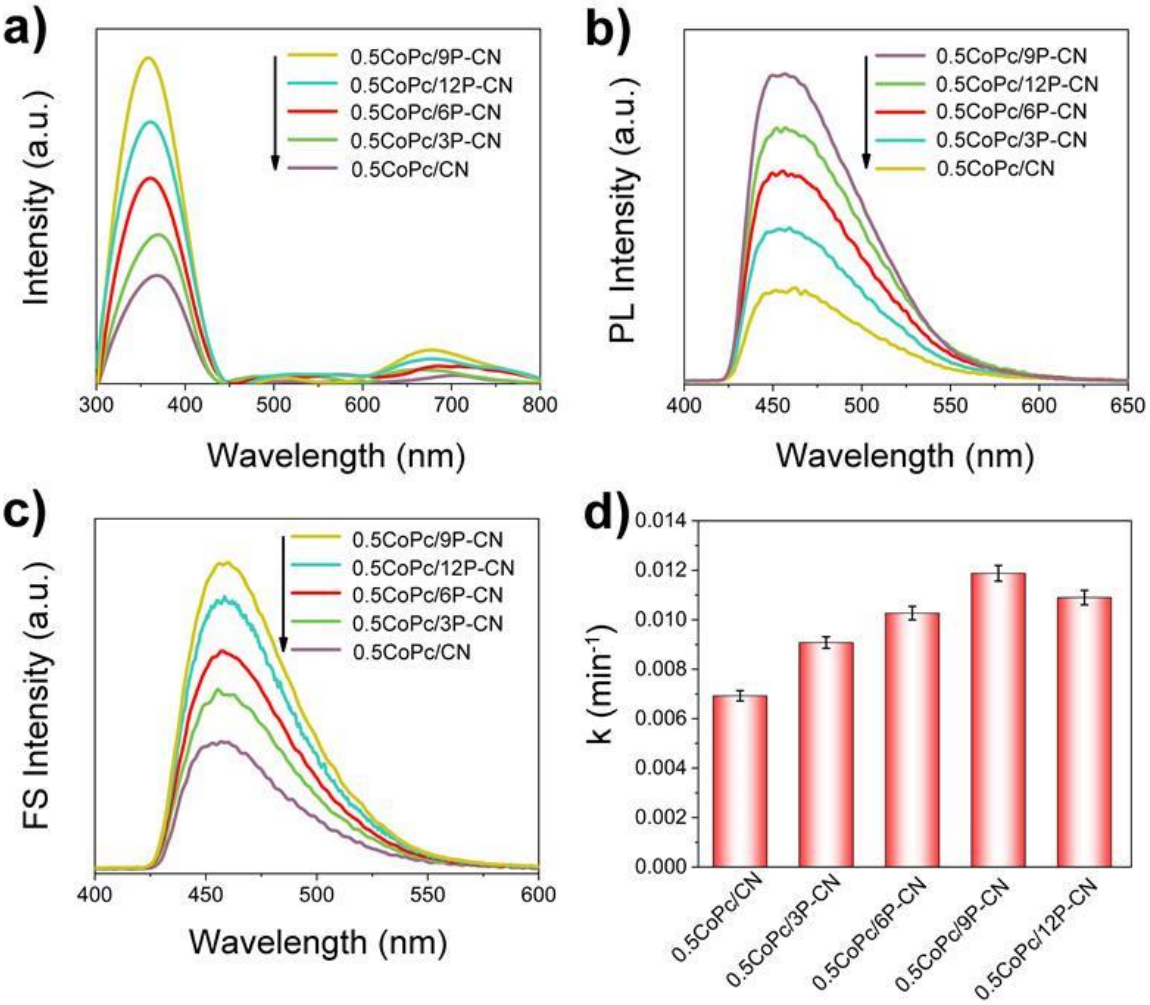

Figure S11. a) SPS spectra, b) PL spectra and c) fluorescence spectra related to the formed hydroxyl radicals under visible-light irradiation of 0.5CoPc/CN and 0.5CoPc/YP-CN. d) Photocatalytic activities for 2,4-DCP degradation of 0.5CoPc/CN and 0.5CoPc/YP-CN under visible-light irradiation. Data are presented as the mean ± SD from three independent experiments.

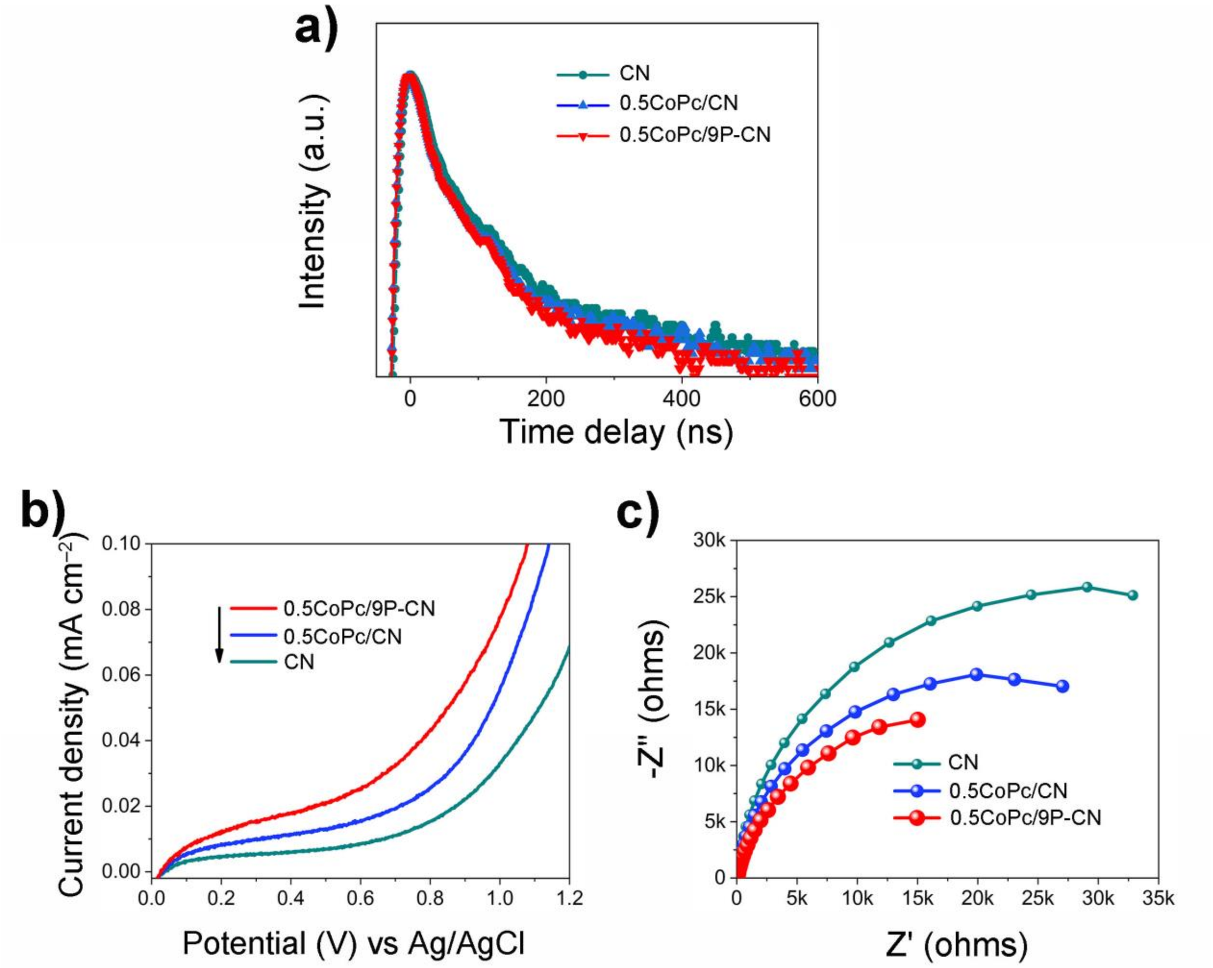

Figure S12. a) The time-resolved PL (TR-PL) spectra, b) photoelectrochemical of linear sweep voltammetric (LSV) scans under visible-light excitation and c) electrochemical impedance spectra (EIS) under visible-light irradiation of CN, 0.5CoPc/CN and 0.5CoPc/9P-CN, respectively.

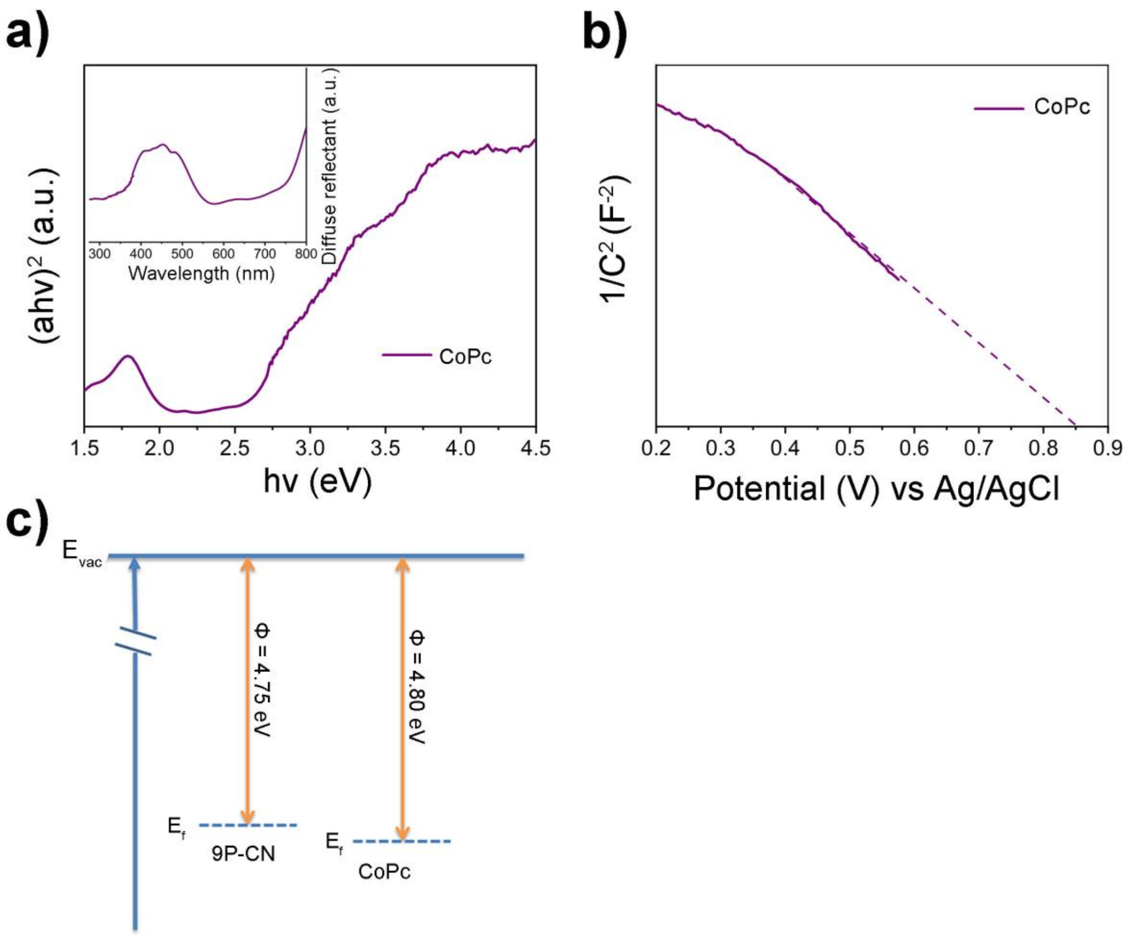

Figure S13. (a) The plots of $(\alpha h\nu)^2$ vs $(h\nu)$ for CoPc. (b) Mott-Schottky plot for CoPc. (c) Schematic work function diagrams of 9P-CN and CoPc.

The optical band gap energy (E_g) of a semiconductor material could be evaluated by the following formula[5]:

$$\alpha h\nu = A(h\nu - E_g)^{n/2}$$

where α, h, ν, A, and E_g represent the absorption coefficient, planck constant, light frequency, proportionality and band gap energy, respectively.

From Figure S13a inset, the E_g value for CoPc is calculated to be 2.44 eV. The Mott-Schottky curve for CoPc was analyzed by the impedance-potential measurements,[6–8] as shown in Figure S13b, which was determined to be approximately +0.85 eV for the HOMO level of CoPc. By the following empirical equations[9,10]:

$$E_{VB}=E_{CB}+E_g$$

The LUMO level of CoPc is calculated to be –1.39 eV. Furthermore, the work functions of 9P-CN and CoPc were shown in Figure S13c.

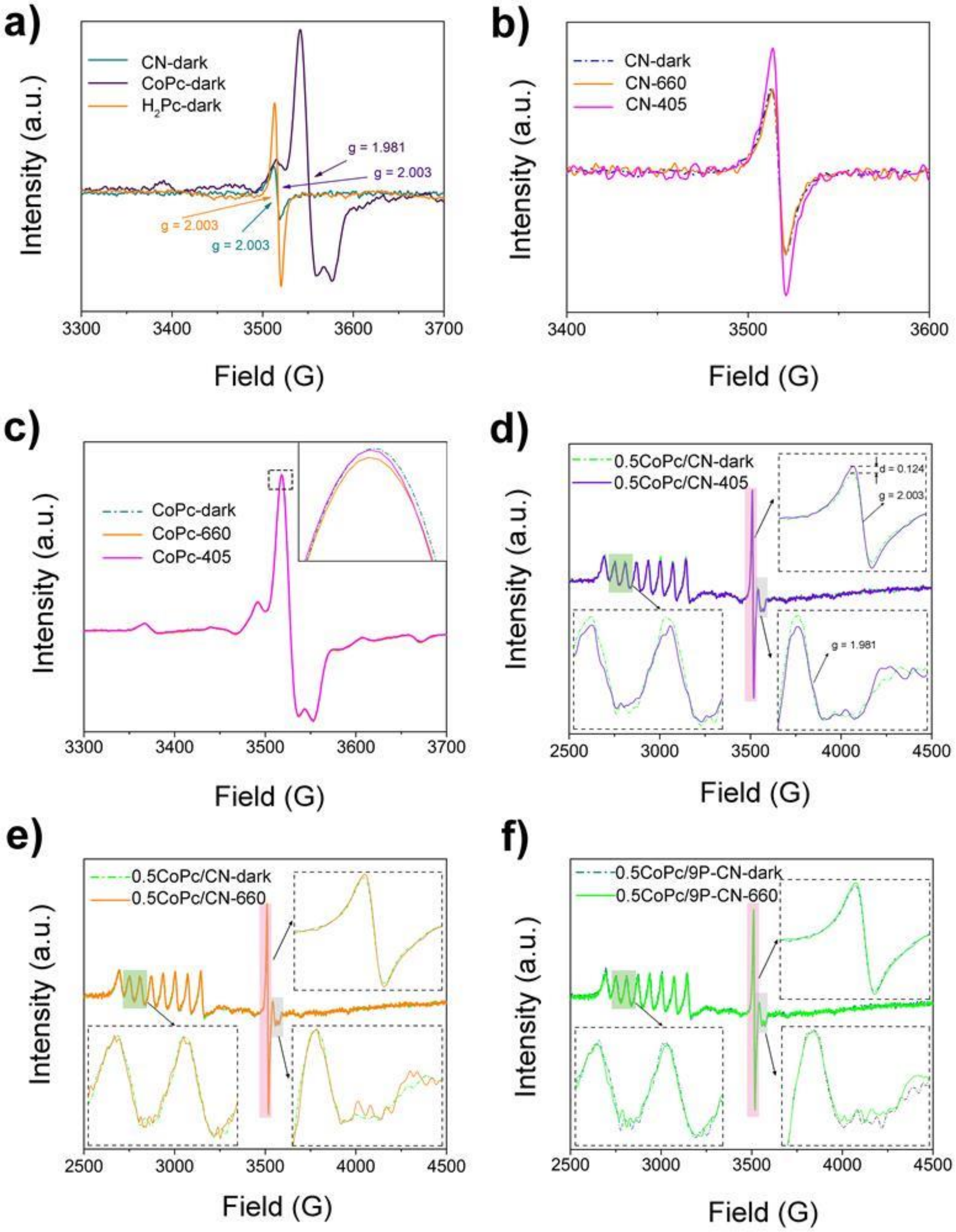

Figure S14. a) EPR spectra of CN, CoPc and H_2Pc, respectively, under dark. b) EPR spectra of CN. c) EPR spectra of CoPc. d) EPR spectra of 0.5CoPc/CN under dark and the irradiation of 405 nm light. e) EPR spectra of 0.5CoPc/CN under dark and the irradiation of 660 nm light. f) EPR spectra of 0.5CoPc/9P-CN under dark and the irradiation of 660 nm light.

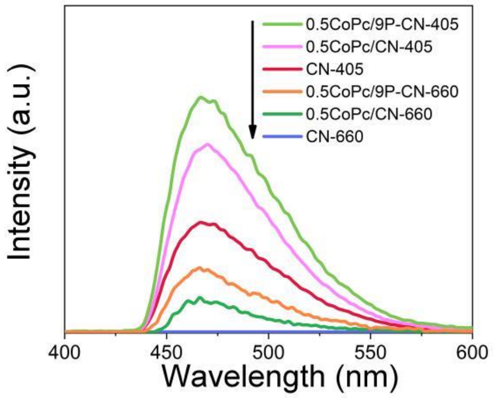

Figure S15. Fluorescence spectra related to the formed hydroxyl radicals of CN, 0.5CoPc/CN and 0.5CoPc/9P-CN under the irradiation of 405 and 660 nm light, respectively.

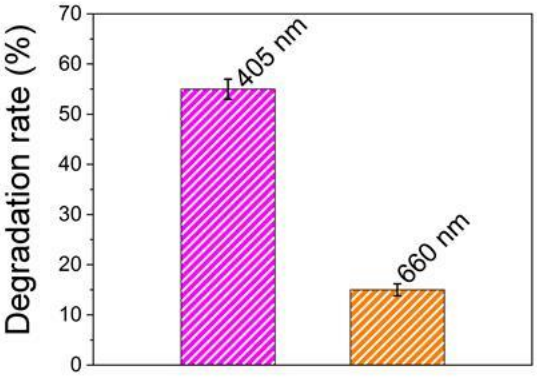

Figure S16. Photocatalytic activities for 2,4-DCP degradation under the irradiation of 405 and 660 nm light, respectively. The light intensities of the light sources 405 nm and 660 nm were normalized the same. Data are presented as the mean ± SD from three independent experiments.

Table S1. Transition energy, transition nature, configurations and coefficients of the excited states and transition characteristics.

Transition energy (eV)	Transition nature	Main configurations and coefficients	Characteristics
1.29	S0→ S13	0.59 (HOMO─2→LUMO)<br>0.23 (HOMO─1→LUMO)	Interfacial charge transfer

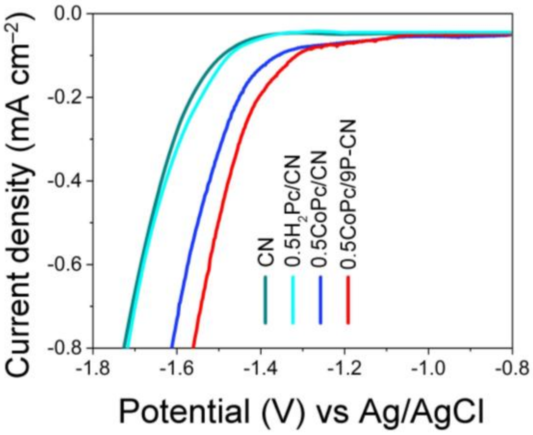

Figure S17. Electrochemical reduction curves of CN, $0.5H_2Pc/CN$, 0.5CoPc/CN, and 0.5CoPc/9P-CN, respectively, with O_2 bubbled.

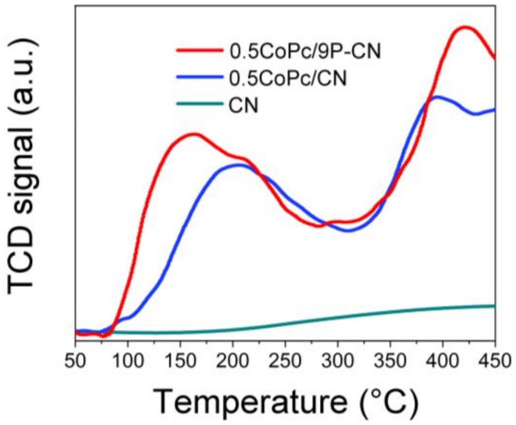

Figure S18. The O_2-TPD curves of CN, 0.5CoPc/CN and 0.5CoPc/9P-CN, respectively.

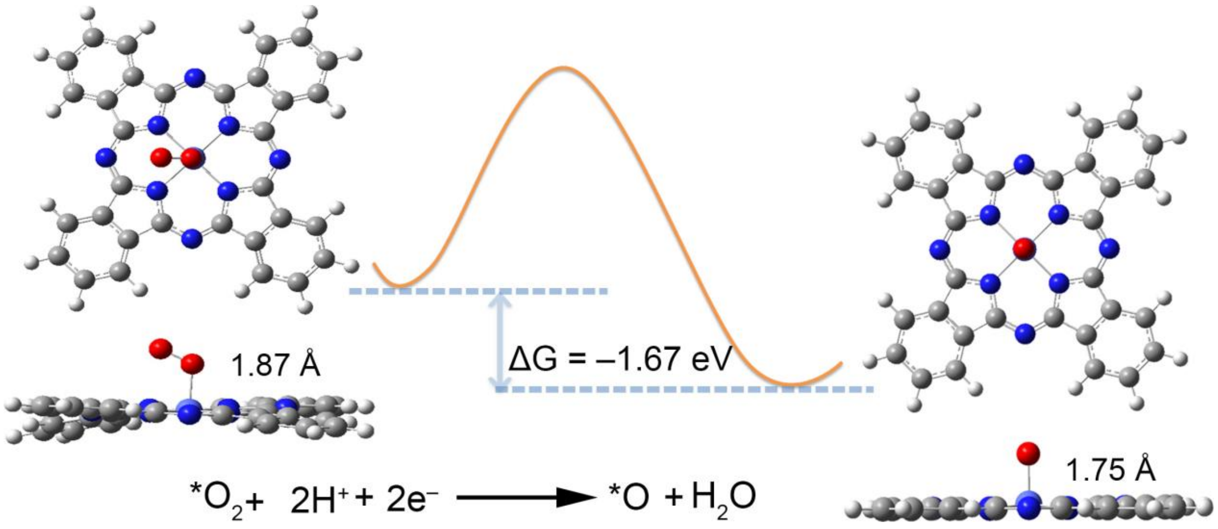

Figure S19. Free energy of *O formation on single-atom Co^{2+} of CoPc and the comparison of Co–O bond length for $*O_2$ and *O adsorption with CoPc, respectively.

a)

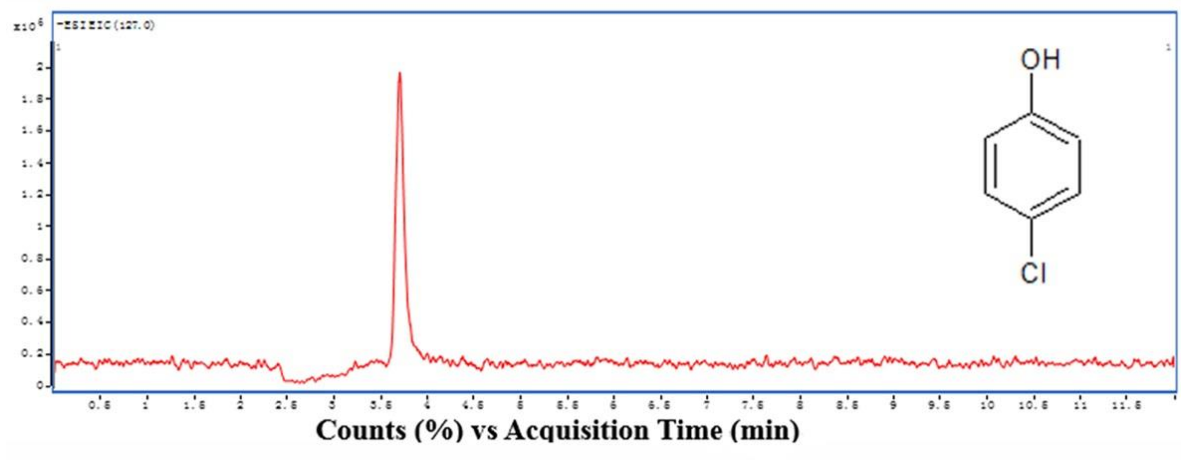
-ESI EIC(127.0)
OH
Cl
Counts (%) vs Acquisition Time (min)

b)

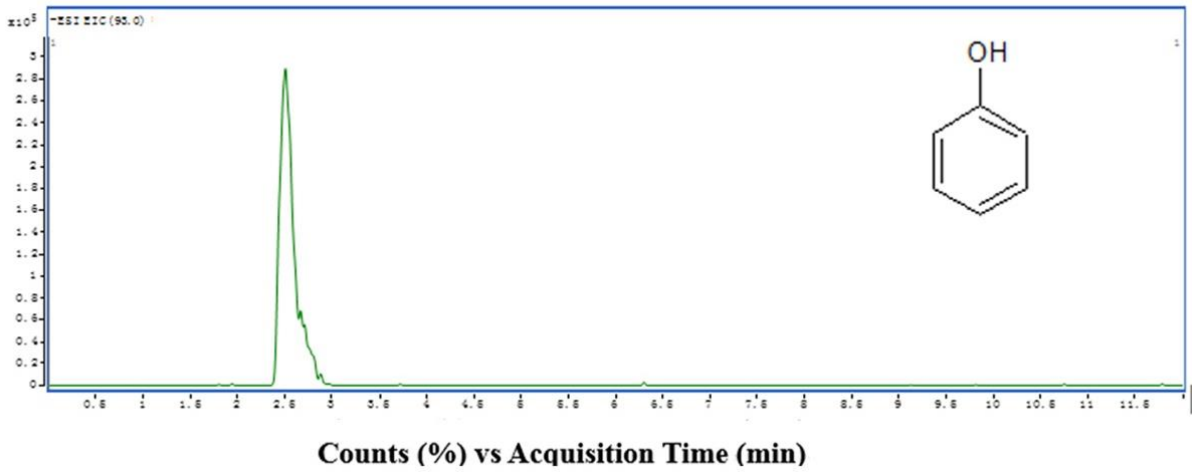
-ESI EIC(93.0)
OH
Counts (%) vs Acquisition Time (min)

c)

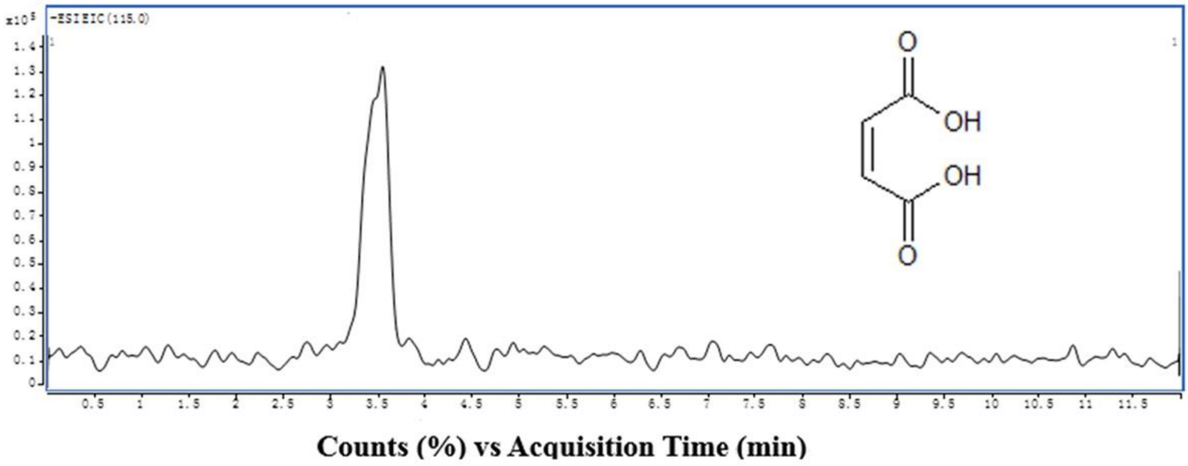
-ESI EIC(115.0)
O
OH
OH
O
Counts (%) vs Acquisition Time (min)

d)

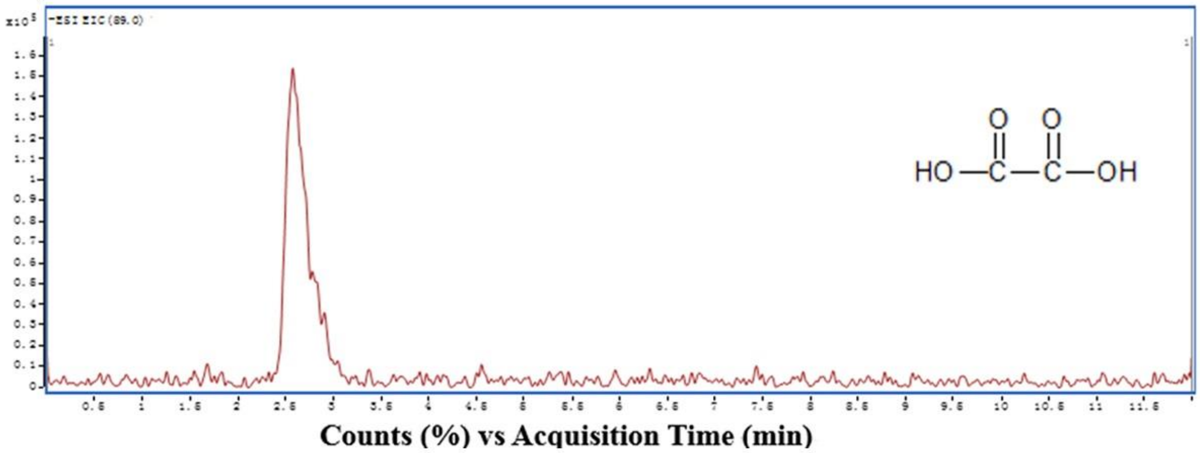

e)

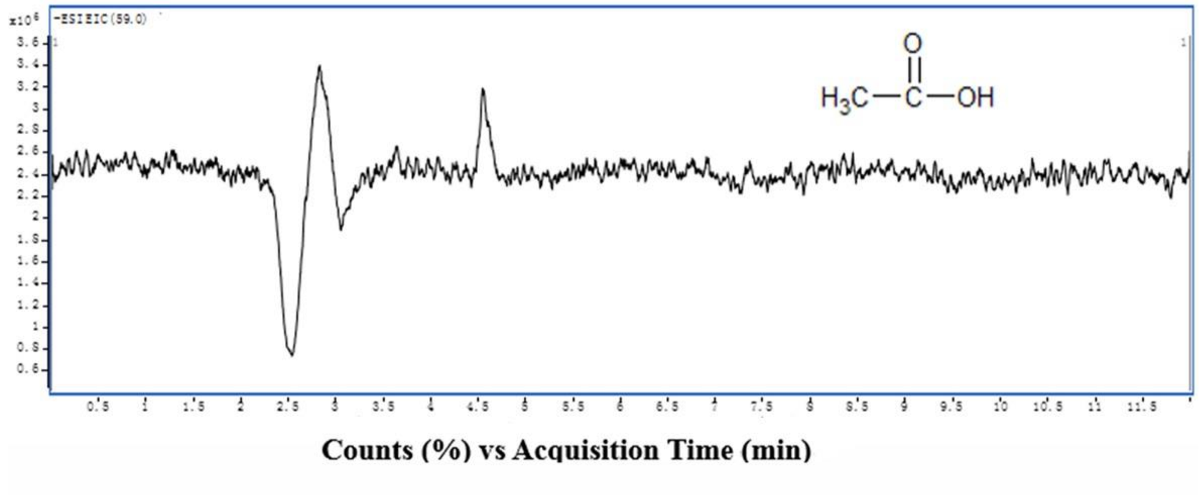

Figure S20. Extract ion chromatographic analyses of reaction intermediates for the degradation of 2,4-DCP over 0.5CoPc/9P-CN under UV–vis light irradiation.

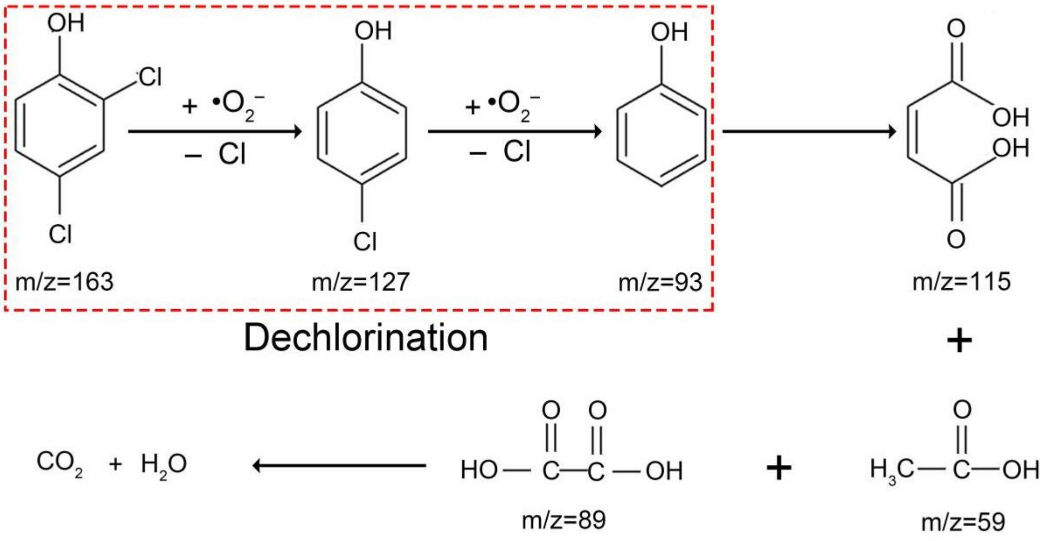

Figure S21. Proposed reaction mechanism for 2,4-DCP degradation under visible-light irradiation with 0.5CoPc/9P-CN as the photocatalyst. In specific, the $•O_2^−$ radicals would directly attack the aromatic ring of 2,4-DCP to remove first $Cl^−$, resulting in parachlorophenol (*m*/*z* value of 127, Figure S20a) then remove the second $Cl^−$ to produce phenol (*m*/*z* = 93, Fig. S20b). Subsequently, the product continues ring-opening cleavage to generate maleic acid (*m*/*z* = 115), oxalic acid (*m*/*z* = 89), acetic acid (*m*/*z* = 59), which are finally mineralized to produce CO_2 and H_2O.

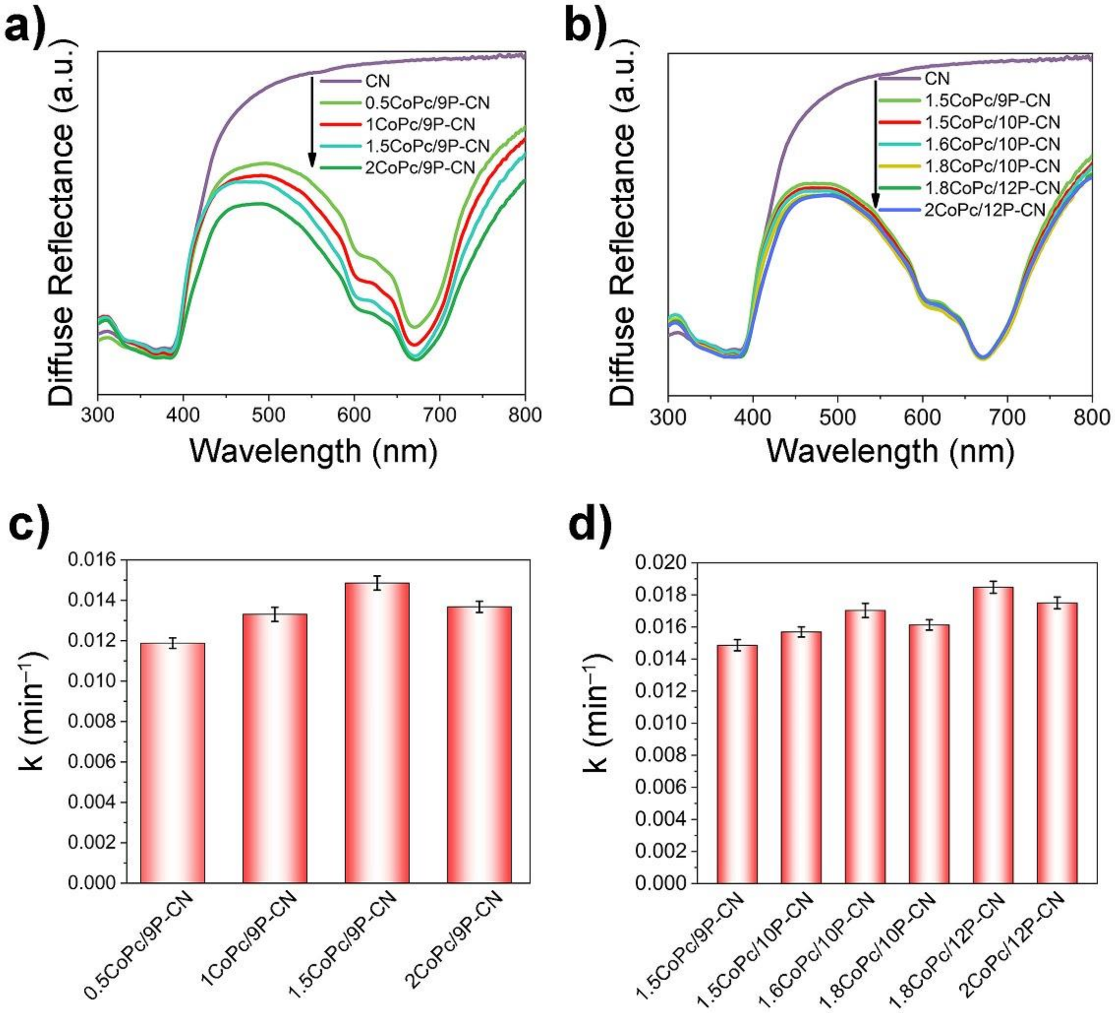

Figure S22. DRS spectra of a) CN and XCoPc/9P-CN and b) CN and XCoPc/YP-CN. c) and d) Corresponding photocatalytic activities for 2,4-DCP degradation under visible-light irradiation. Data are presented as the mean ± SD from three independent experiments.

Table S2. Contrast with supported MPc photocatalysts.

Entry	Photocatalyst	Interfacial interaction mode between MPc and support	Clarification of dual charge separation modes[a]	Clarification of photoactivity contribution corresponding to charge separation mode	Clarification of the catalytic effect of M center in MPc	Reaction type	Ref.
1	g-C_3N_4-INA-$FePcCl_{16}$	Axial coordination	X	X	√	CBZ[b] degradation	[11]
2	ZnPc/C_3N_4	Unclarified	X	X	√	CO_2 reduction	[12]
3	g-C_3N_4-IMA-$FePcCl_{16}$	Axial coordination	X	X	X	CBZ degradation	[13]
4	ZnPc/PCN	Unclarified	X	X	X	H_2 production	[14]
5	LI-4/g-C_3N_4/Zn-tri-PcNc	Adsorption	X	X	X	H_2 Production	[15]
6	mpg-CN_x/ CoPPc	π–π Stacking	X	√	√	CO_2 reduction	[16]
7	g-C_3N_4/ZnTcPc/GQDs	Covalent	X	X	X	RhB[c] degradation	[17]
8	CoPc/TiO_2	Covalent	X	X	X	CO_2 conversion	[18]
9	ACFs-CoPc/PMS	Covalent	X	X	√	AR1[d] degradation	[19]
10	CoPc/NiO	Covalent	X	X	X	CO_2 reduction	[20]
11	Zn-tri-PcNc/g-C_3N_4	Unclarified	X	X	X	H_2 Production	[21]
12	1.8CoPc/12P-CN	H-bonding	√	√	√	2,4-DCP degradation Benzyl alcohol oxidation	Our work

Note: a) Photoelectrons from MPc to support versus from support to MPc. b) CBZ is short for carbamazepine. c) RhB is short for rhodamine B. d) AR1 is short for acid red 1.

Five indexes (first row and column 3−7 of Table S2) have been set for evaluating the scientific depth of individual work. In terms of the interfacial interaction between MPc and support material, most works have not clarified or evidenced the specific interactions and residue works have adopted the covalent-bonding to load MPc (entry 7−10), which synthetic processes are relatively complex. Noteworthily, our work is the first one to clearly validate the H-bonding interfacial interaction, especially which has been achieved by a facile phosphate-induction resulting in a high dispersion of CoPc. Secondly, in terms of the investigation on the photocatalytic mechanism, among all works our one has firstly clearly clarified the dual charge separation modes with the support of experimental evidences like EPR results, etc as well as DFT calulations. Furthermore, the photoactivity contribution corresponding to the charge separation mode has been individually illustrated. More important, few works focus on utilizing the single metal centers in MPc as the catalytic sites and only in our work the single-atom Co-N_4 (II) sites were highlighted and well studied by EPR and DFT calculations. Finally, what's the most unique aspect of our work is the different application, which could be found the photocatalytic O_2 activation especially the successful aerobic selective alcohol oxidation has been realized for the first time among similar photocatalytic systems. Based above, it's concluded that among similar photocatalytic systems our work is against the grain and has achieved deeper scientific recognization.

Table S3. Contrast with CN-based photocatalysts for aerobic selective oxidation of benzyl alcohol.

Entry	Photocatalyst	Reaction conditions	Benzyl alcohol (mmol)	Sel. (%)[a]	Yield (%)	Ref.
1	VO@g-C_3N_4 (25 mg)	40 W domestic bulb, H_2O_2 (1.5 mmol), acetonitrile solvent (2 mL),1 h	1	-	98	[22]
2	mpg-C_3N_4 (50 mg)	300 W Xe Lamp (λ > 420 nm), trifluorotoluene solvent (9 mL), O_2 (0.8 MPa), 100 °C, 3 h	1	99	57	[23]
3	$BiVO_4$/g-C_3N_4 (5/5) (20 mg)	250 W visible light illumination (λ > 400 nm), acetonitrile (8 ml), O_2, 25-27 °C, 16 h	0.35	77.3	81.3	[24]
4	1.8CoPc/12P-CN (50 mg)	300 W Xe Lamp, acetonitrile solvent (5 mL), O_2, 30 °C, 6 h	0.2	99	25.6	Our work

Note: a) Selectivity towards the main product benzylaldehyde.

For further highlighting the advancement of our work, we have compared the photocatalytic performance with reported CN-based ones for the aerobic oxidation of benzyl alcohol as the benchmarked reaction and shown in Table S3. Noteworthily, without additives like H_2O_2 as entry 1, or high temperature and high O_2 pressure like entry 2, 1.8CoPc/12P-CN still showed impressive benzyl aldehyde yield, which also has higher selectivity over entry 3. Therefore, for CN based photocatalysts for aerobic alcohol oxidation, CoPc/P-CN photocatalysts have exhibited great potential to achieve highly active and selective conversion under room temperature as well as atmospheric pressure.

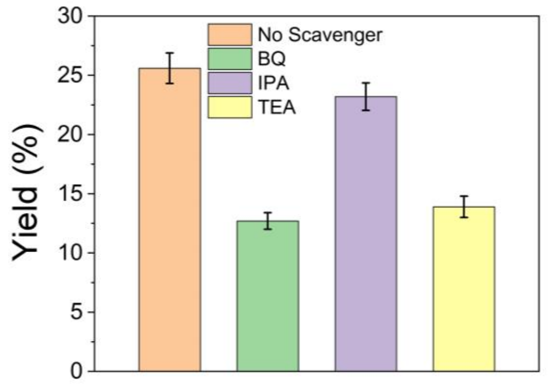

Figure S23. Photocatalytic activities for benzyl alcohol conversion of 1.8CoPc/12P-CN in the presence of the different scavengers. Data are presented as the mean ± SD from three independent experiments.

3. References

[1] J. D. Chai and M. H. Gordon, *Phys. Chem. Chem. Phys*. **2008**, *10*, 6615.

[2] B. Barone, G. Mennucci, H. Petersson, Gaussian 09, *Gaussian. Inc. Wallingford*, CT **2009**.

[3] Y. T. Xiao, G. H. Tian, W. Li, Y. Xie, B. J. Jiang, C. G. Tian, D. Y. Zhao, H. G. Fu, *J. Am. Chem. Soc.* **2019**, *141*, 2508.

[4] Z. -A. Lan, G. G. Zhang, X. C. Wang, *Appl. Catal., B* **2016**, *192*, 116.

[5] T. Y. Shi, H. N. Li, L. J. Ding, F. H. You, L. Ge, Q. Liu and K. Wang, *ACS Sustainable Chem. Eng.* **2019**, *7*, 3319.

[6] S. P. Adhikari, Z. D. Hood, H. Wang, R. Peng, A. Krall, H. Li, V. W. Chen, K. L. More, Z. L. Wu, S. Geyer, A. Lachgar, *Appl. Catal., B* **2017**, *217*, 448.

[7] L. J. Ye, D. Wang and S. J. Chen, *ACS Appl. Mater. Interfaces,* **2016**, *8*, 5280.

[8] S. A. Ni, F. J. Han, W. Wang, D. F. Han, Y. Bao, D. X. Han, H. Y. Wang and L. Niu, *Sens. Actuators B* **2018**, *259*, 963.

[9] F. Chen, Q. Yang, Q. Wang, F. B. Yao, J. Sun, Y. L. Wang, C. Zhang, X. M. Li and C. G. Niu, *Appl. Catal., B* **2017**, *209*, 493.

[10] Y. Z. Hong, Y. H. Jiang, C. S. Li, W. Q. Fan, X. Yan, M. Yan and W. D. Shi, *Appl. Catal., B* **2016**, *180*, 663.

[11] F. Wu, H. W. Huang, T. F. Wu, W. Y. Lu, N. Li, W. X. Chen, *Appl. Catal., B* **2017***, 218,* 230.

[12] J. G. Zhang, X. J. Li, Y. H. Qin, S. Q. Zhang, M. S. Sun, X. G. Duan, H. Q. Sun, P. Q. Li, S. B. Wang, *J. Catal.* **2019**, *371*, 214.

[13] L. L. Dong, T. F. Xu, W. X. Chen, W. Y. Lu, *Chemical Engineering Journal* **2019**, *357*, 198.

[14] P. Zeng, J. M. Wang, Y. Y. Guo, R. J. Li, G. Q. Mei, T. Y. Peng, *Chemical Engineering Journal* **2019**, *373*, 651.

[15] X. H. Zhang, T. Y. Peng, L. J. Yu, R. J. Li, Q. Q. Li, Z. Li, *ACS Catal.* **201**5, *5*, 504.

[16] S. Roy, E. Reisner, *Angew. Chem.* **2019**, *131*, 1.

[17] T. F. Xu, D. N. Wang, L. L. Dong, H. B. Shen, W. Y. Lu, W. X. Chen, *Appl. Catal., B* **2019**, *244*, 96.

[18] P. K. Prajapati, A. Kumar, S. L. Jain, *ACS Sustainable Chem. Eng.* **2018**, *6*, 7799.

[19] Z. F. Huang, H. W. Bao, Y. Y. Yao, W. Y. Lu, W. X. Chen, *Appl. Catal., B* **2014**, *154*, 36.

[20] X. H. Zhang, L. J. Yu, C. S. Zhuang, T. Y. Peng, R. J. Li, X. G. Li, *ACS Catal.* **2014**, *4*, 162.

[21] P. K. Prajapatia, H. Singh, R. Yadav, A. K. Sinha, S. Szuneritsd, R. Boukherroubd, S. L. Jaina, *Applied Surface Science* **2019**, *467*, 370.

[22] S. Verma, R. B. N. Baig, M. N. Nadagouda, R. S. Varma, *ACS Sustainable Chem. Eng.* **2016**, *4*, 1094.

[23] F. Z. Su, S. C. Mathew, G. Lipner, X. Z. Fu, M. Antonietti, S. Blechert, X. C. Wang, *J. Am. Chem. Soc.* **2010**, *132*, 16299.

[24] S. Samanta, S. Khilari, D. Pradhan, R. Srivastava, *ACS Sustainable Chem. Eng.* **2017**, *5*, 2562.
